# Supplementary material for: Discovering a New Okadaic Acid Derivative, a Potent HIV Latency Reversing Agent from Prorocentrum lima PL11: Isolation, Structural Modification, and Mechanistic Study
Source: Mar Drugs. 2023 Feb 27;21(3):158. doi: 10.3390/md21030158 (PMC10058201; doi:10.3390/md21030158)

Supporting Information

Discovering a New Okadaic Acid Derivative as Potent HIV Latency-Reversing Agent from *Prorocentrum lima* PL11: Isolation, Structural Modification, and Mechanistic Study

Dong Huang[1,#], Lian-Shuai Ding[2,4,#], Fang-Yu Yuan[1], Shu-Qi Wu[1], Han-Zhuang Weng[1], Xiao-Qing Tian[3], Gui-Hua Tang[1], Cheng-Qi Fan[3,*], Xiang Gao[2,*] and Sheng Yin[1,*]

[1] School of Pharmaceutical Sciences, Southern Marine Science and Engineering Guangdong Laboratory (zhuhai), Sun Yat-sen University, Guangzhou, Guangdong 510006, China

[2] State Key Laboratory of Cellular Stress Biology, School of Pharmaceutical Sciences, Xiamen University, Xiamen, Fujian 361102, China

[3] East China Sea Fisheries Research Institute, Chinese Academy of Fishery Sciences, Shanghai 200090, China

[4] School of life Sciences, Xiamen University, Xiamen, Fujian 361102, China;

[#] D. Huang and L.-S. Ding contributed equally to this work

* Correspondence: yinsh2@mail.sysu.edu.cn (S. Yin); xgao@xmu.edu.cn (X. Gao); fancq@ecsf.ac.cn (C-Q. Fan)

Contents

Figure S1. The HIV-latency-reversing activity of OA derivates **2**–**6** at 10 μM measured in 2D10 cell.

Figure S2. The HIV-latency-reversing activity of compound **7** (EC$_{50}$) measured in 2D10 cell.

2. NMR, HRESIMS, and IR spectra of 1–7

Figure S3. ^{1}H NMR spectrum of **1** in $CDCl_3$.

Figure S4. ^{13}C NMR and DEPT 135 spectra of **1** in $CDCl_3$.

Figure S5. ^{1}H NMR spectrum of **2** in $CDCl_3$.

Figure S6. ^{13}C NMR and DEPT 135 spectra of **2** in $CDCl_3$.

Figure S7. ^{1}H NMR spectrum of **3** in $CDCl_3$.

Figure S8. ^{13}C NMR and DEPT 135 spectra of **3** in $CDCl_3$.

Figure S9. HSQC spectrum of **3** in $CDCl_3$.

Figure S10. HMBC spectrum of **3** in $CDCl_3$.

Figure S11. HRESIMS spectrum of **3**.

Figure S12. IR (KBr disc) spectra of **3**.

Figure S13. ^{1}H NMR spectrum of **4** in $CDCl_3$.

Figure S14. ^{13}C NMR and DEPT 135 spectra of **4** in $CDCl_3$.

Figure S15. HSQC spectrum of **4** in $CDCl_3$.

Figure S16. HMBC spectrum of **4** in $CDCl_3$.

Figure S17. ^{1}H–^{1}H COSY spectrum of **4** in $CDCl_3$.

Figure S18. HRESIMS spectrum of **4**.

Figure S19. IR (KBr disc) spectra of **4**.

Figure S20. ^{1}H NMR spectrum of **5** in $CDCl_3$.

Figure S21. ^{13}C NMR and DEPT 135 spectra of **5** in $CDCl_3$.

Figure S22. HSQC spectrum of **5** in $CDCl_3$.

Figure S23. HMBC spectrum of **5** in $CDCl_3$.

Figure S24. ^{1}H–^{1}H COSY spectrum of **5** in $CDCl_3$.

Figure S25. HRESIMS spectrum of **5**.

Figure S26. IR (KBr disc) spectra of **5**.

Figure S27. ^{1}H NMR spectrum of **6** in $CDCl_3$.

Figure S28. ^{13}C NMR and DEPT 135 spectra of **6** in $CDCl_3$.

Figure S29. HSQC spectrum of **6** in $CDCl_3$.

Figure S30. HMBC spectrum of **6** in $CDCl_3$.

Figure S31. ^{1}H–^{1}H COSY spectrum of **6** in $CDCl_3$.

Figure S32. HRESIMS spectrum of **6**.

Figure S33. IR (KBr disc) spectra of **6**.

Figure S34. ^{1}H NMR spectrum of **7** in $CDCl_3$.

Figure S35. ^{13}C NMR and DEPT 135 spectra of **7** in $CDCl_3$.

Figure S36. HSQC spectrum of **7** in $CDCl_3$.

Figure S37. HMBC spectrum of **7** in $CDCl_3$.

Figure S38. ^{1}H–^{1}H COSY spectrum of **7** in $CDCl_3$.

Figure S39. HRESIMS spectrum of **7**.

Figure S40. IR (KBr disc) spectra of **7**.

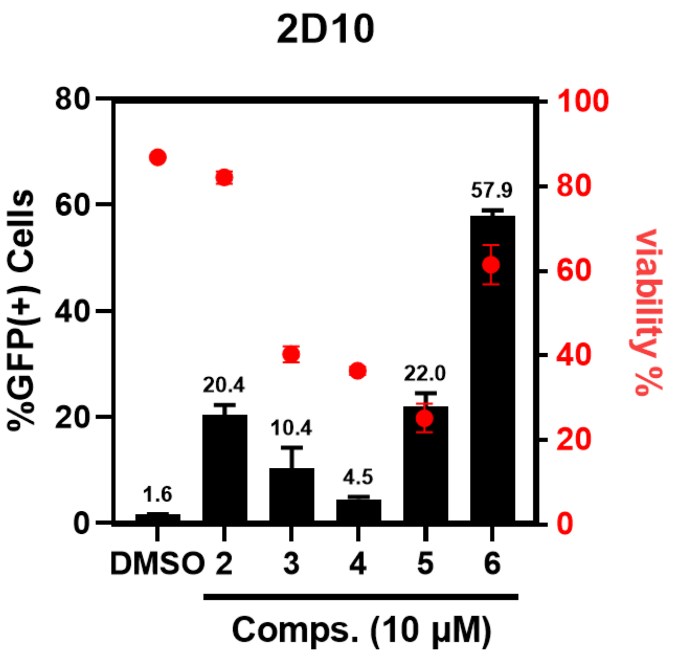

Figure S1. The HIV-latency-reversing activity of OA derivates **2–6** at 10 μM measured in 2D10 cell. Cell viability was labeled as red dots. Black bars represented GFP expression.

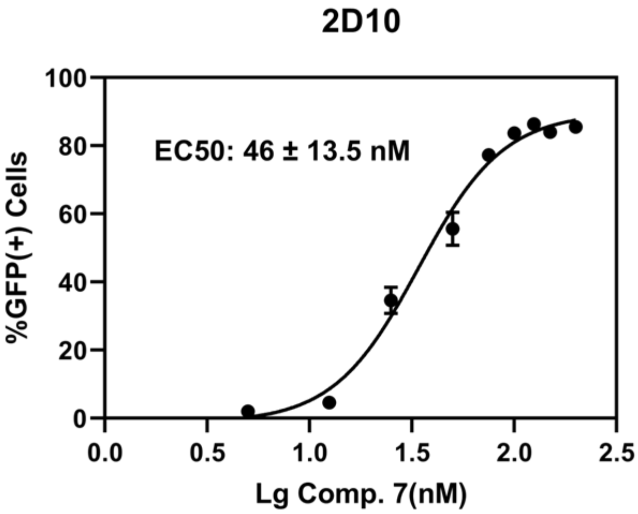

Figure S2. The HIV-latency-reversing activity of compound **7** (EC$_{50}$) measured in 2D10 cell.

2. NMR, HRESIMS, and IR spectra of 1–7.

Figure S3. ^{1}H NMR spectrum of **1** in $CDCl_3$.

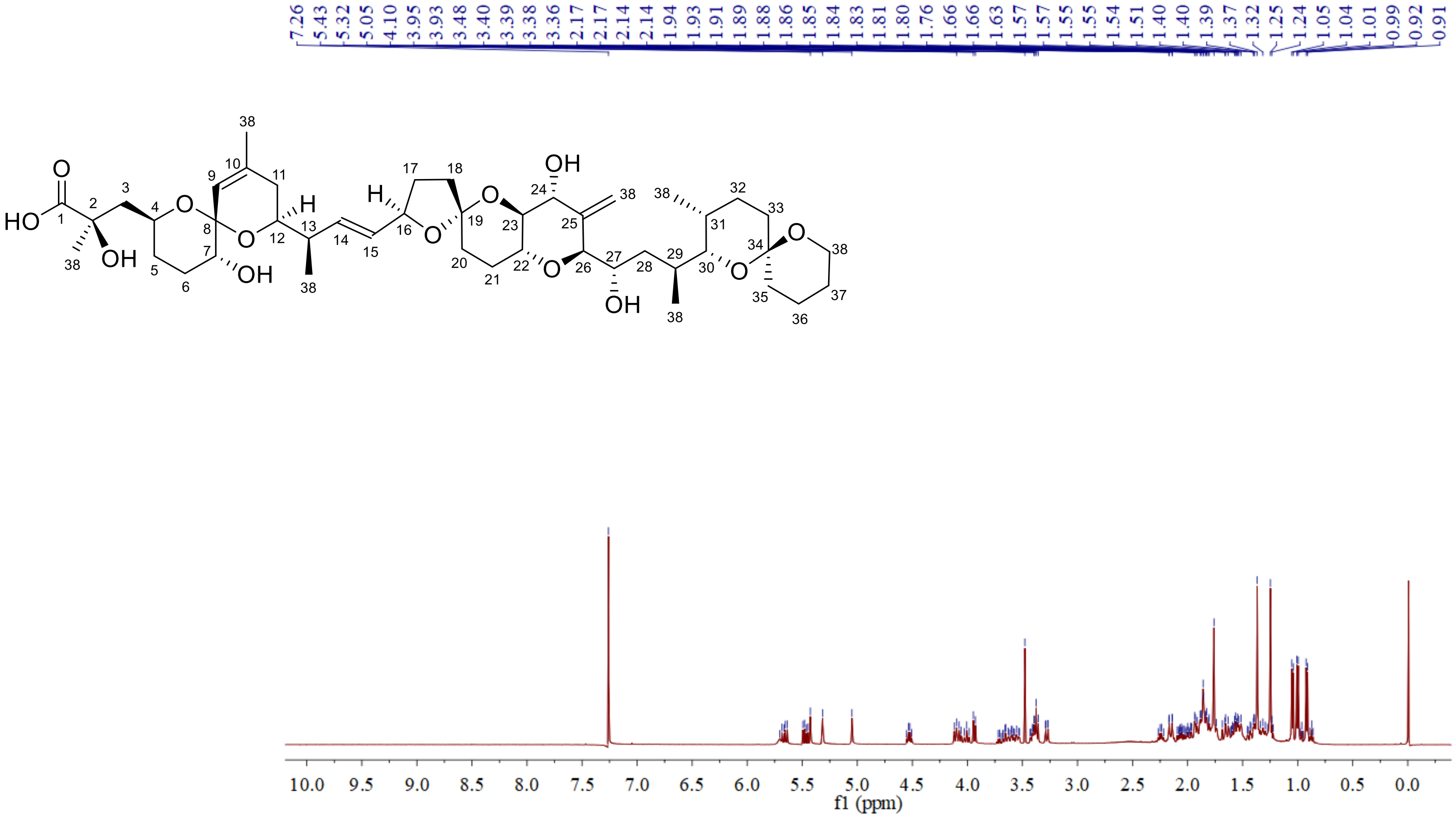

Figure S4. ^{13}C NMR and DEPT 135 spectra of **1** in $CDCl_3$.

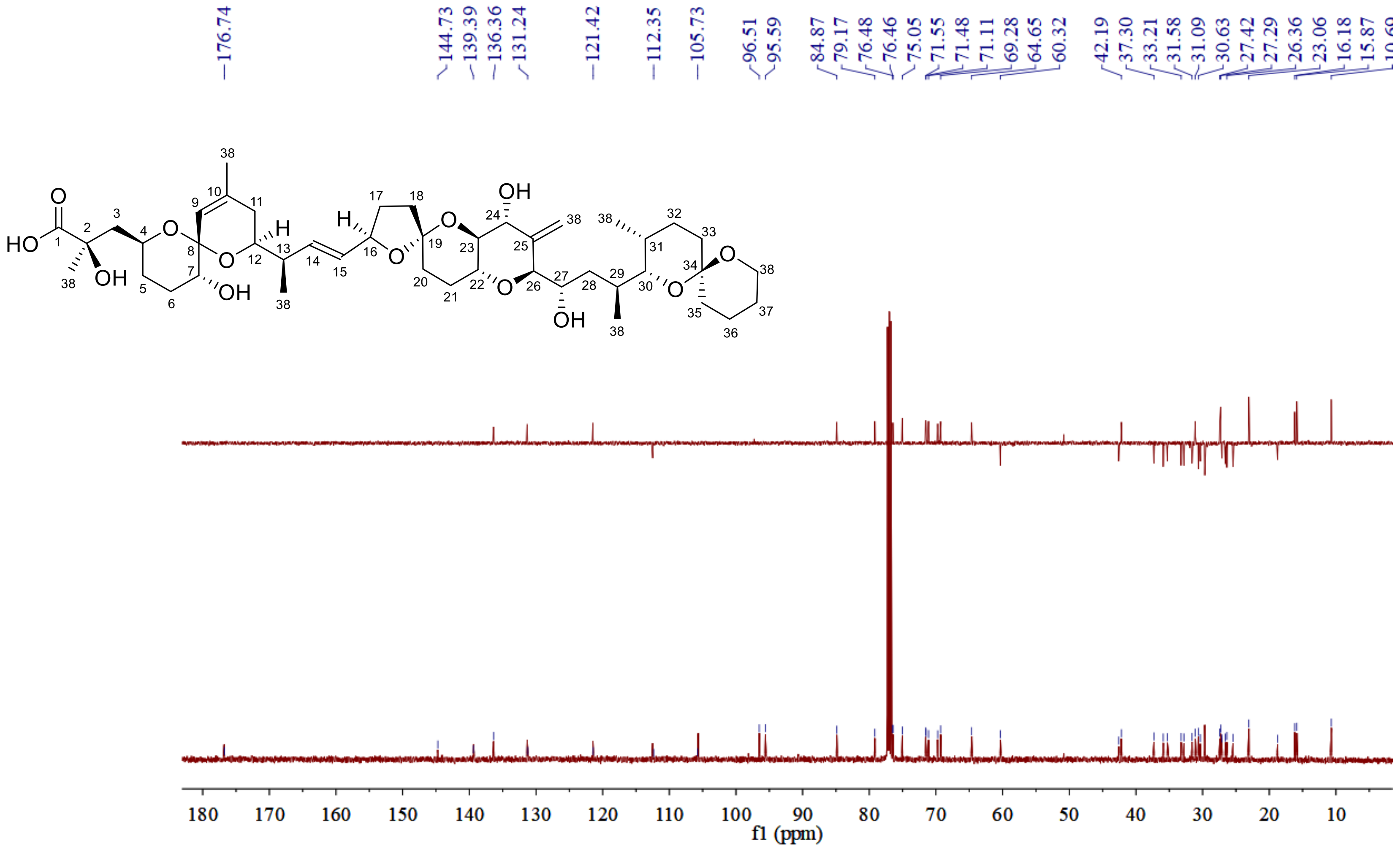

Figure S5. ^{1}H NMR spectrum of **2** in $CDCl_3$.

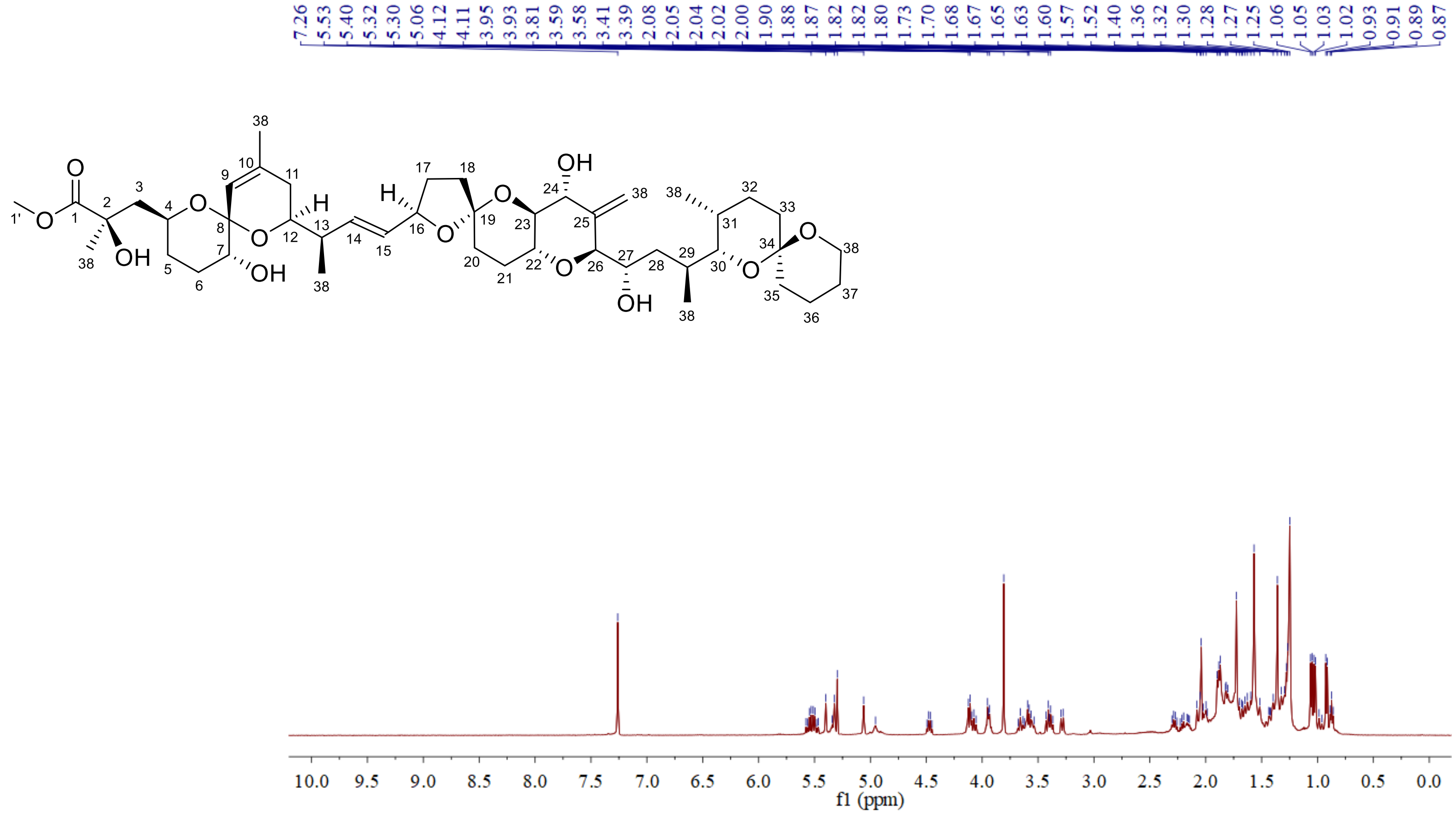

Figure S6. ^{13}C NMR and DEPT 135 spectra of **2** in $CDCl_3$.

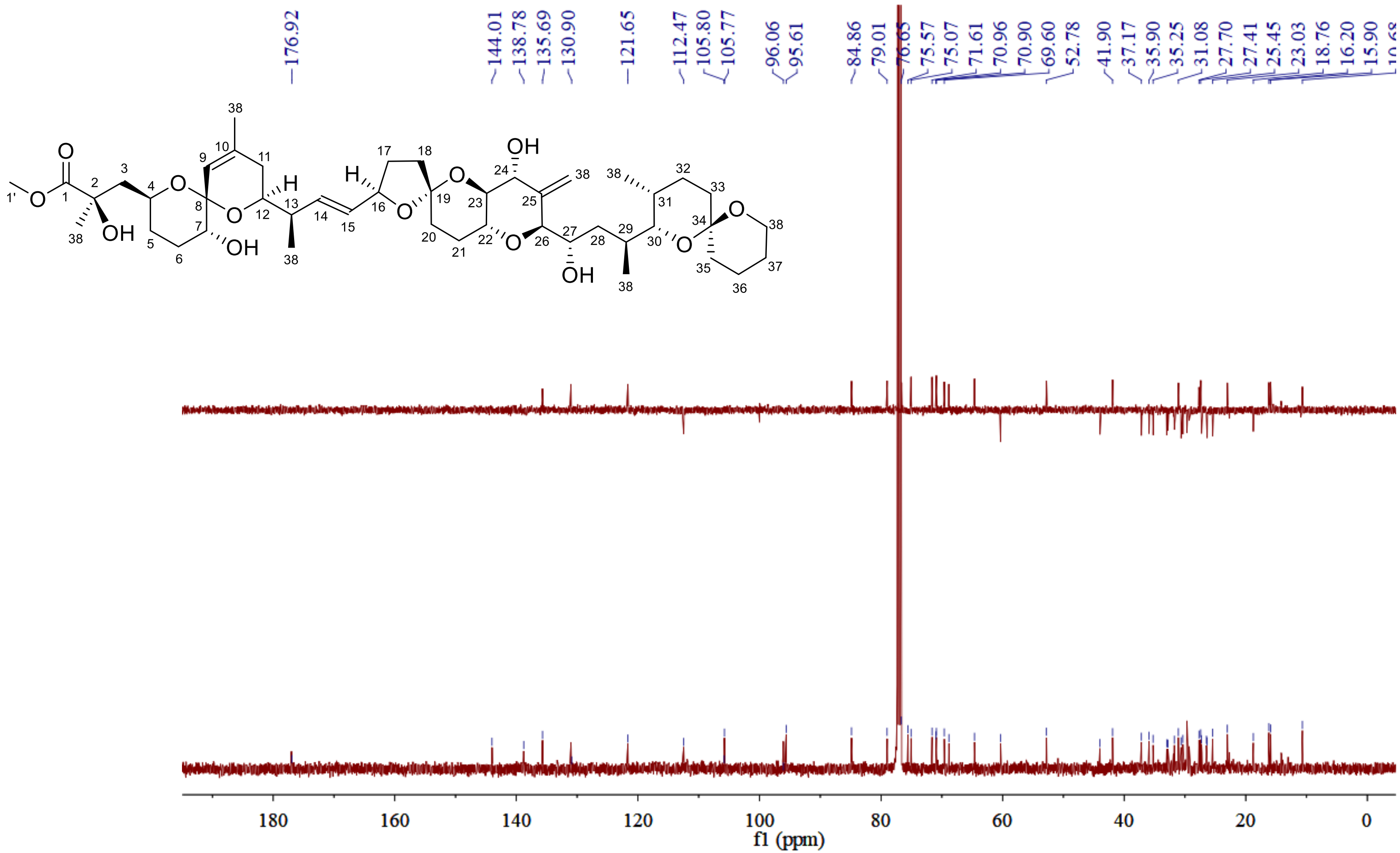

Figure S7. ^{1}H NMR spectrum of **3** in $CDCl_3$.

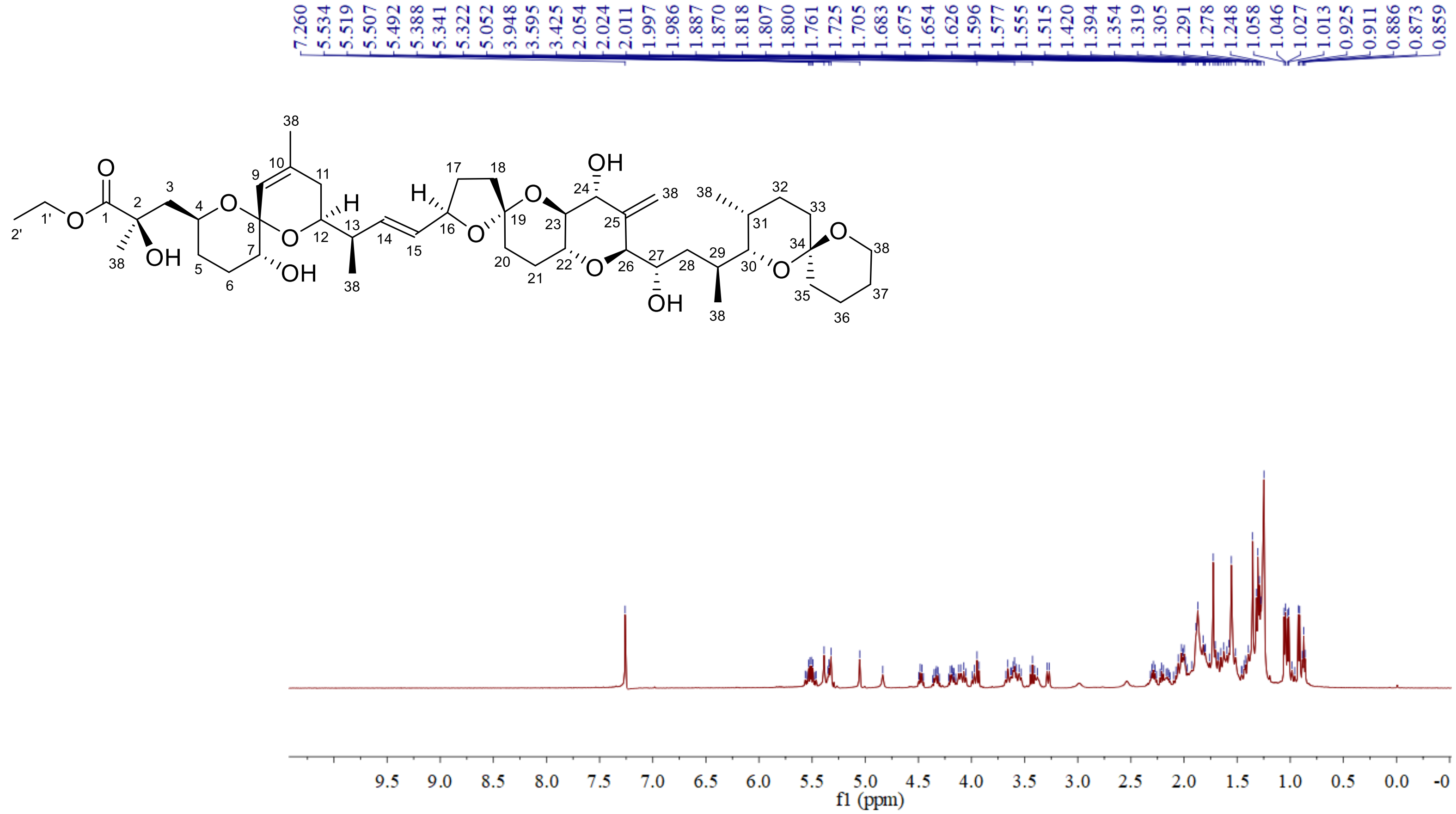

Figure S8. ^{13}C NMR and DEPT 135 spectra of **3** in $CDCl_3$.

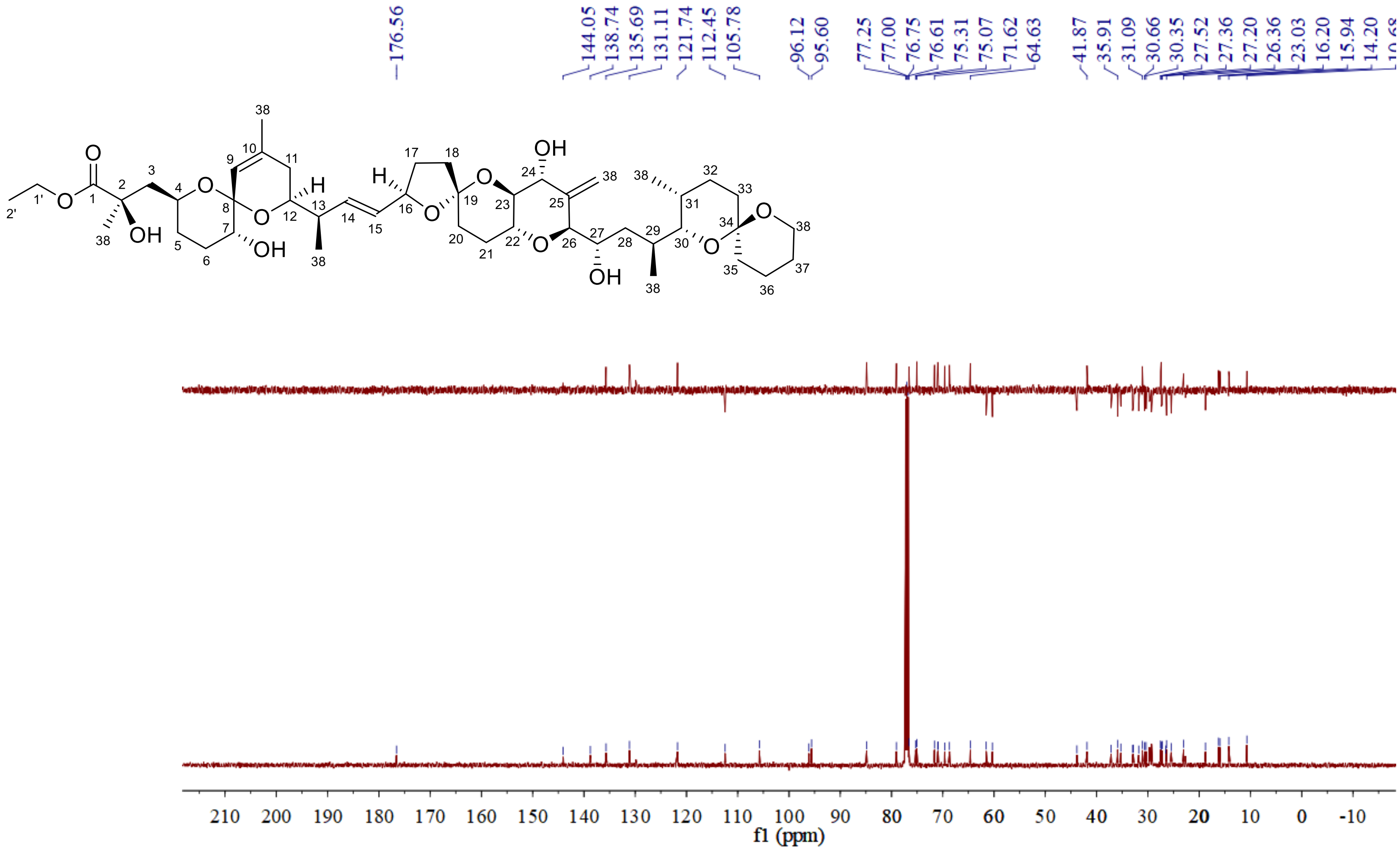

Figure S9. HSQC spectrum of **3** in $CDCl_3$.

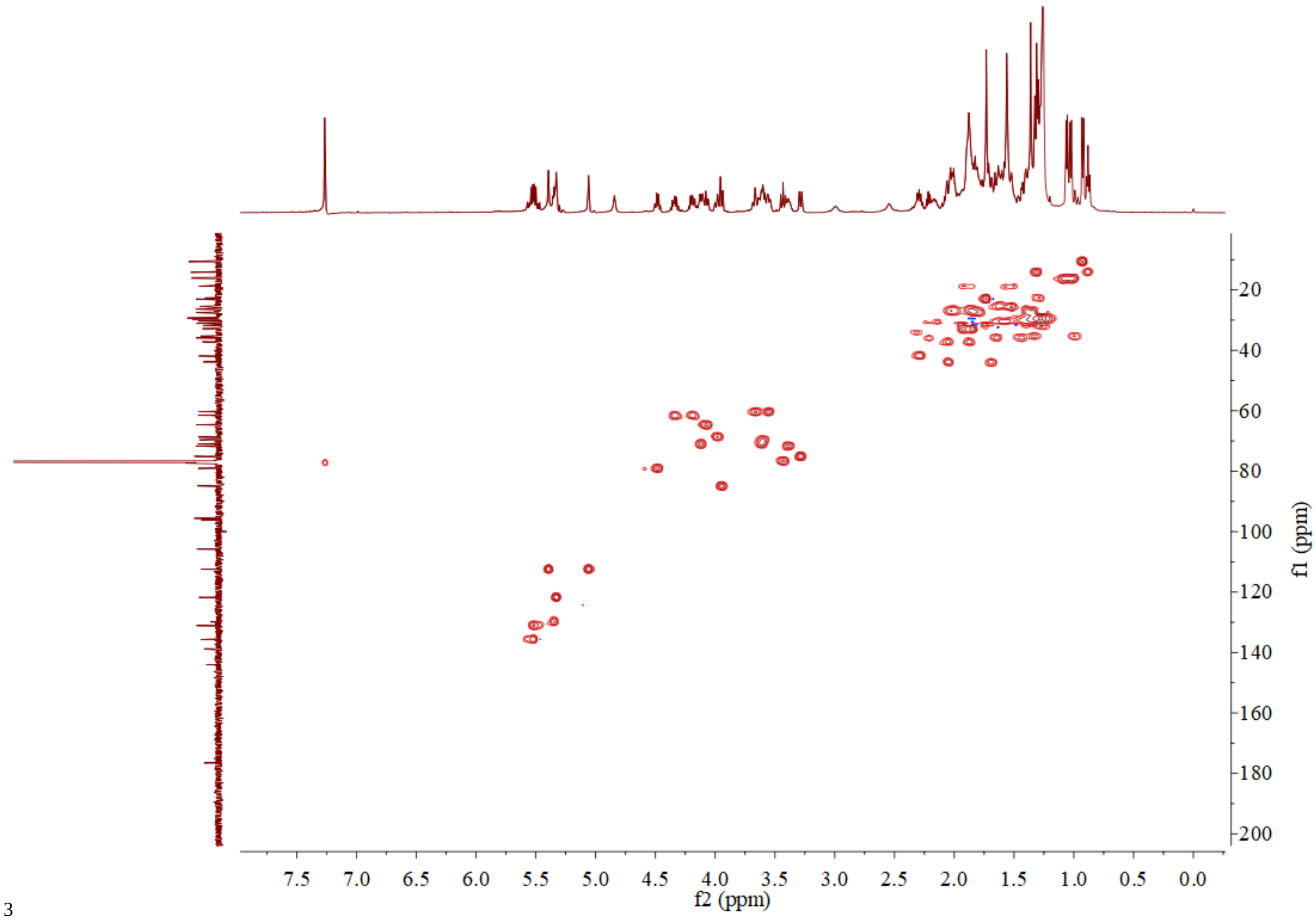

3

Figure S10. HMBC spectrum of **3** in $CDCl_3$.

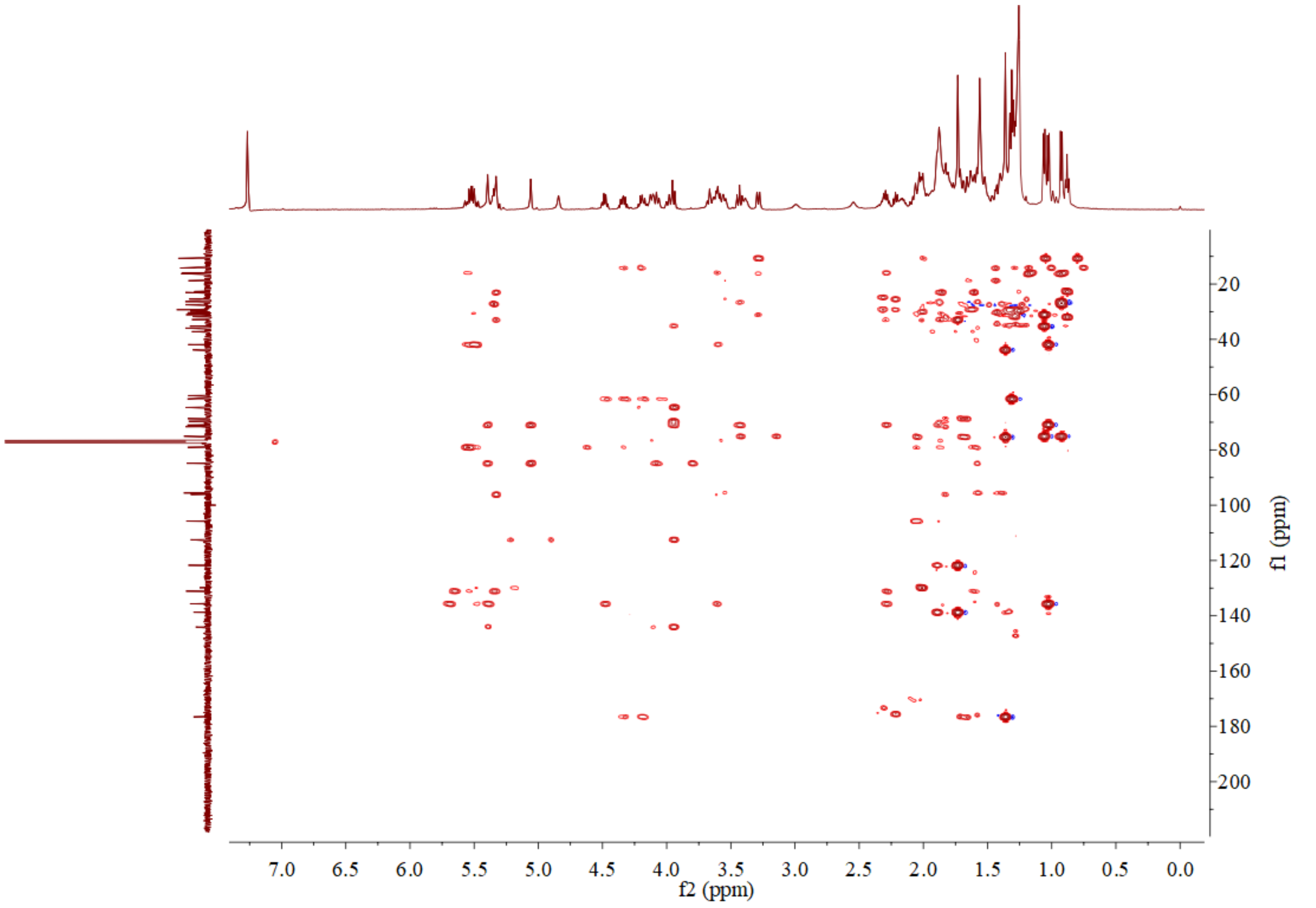

Figure S11. HRESIMS spectrum of **3**.

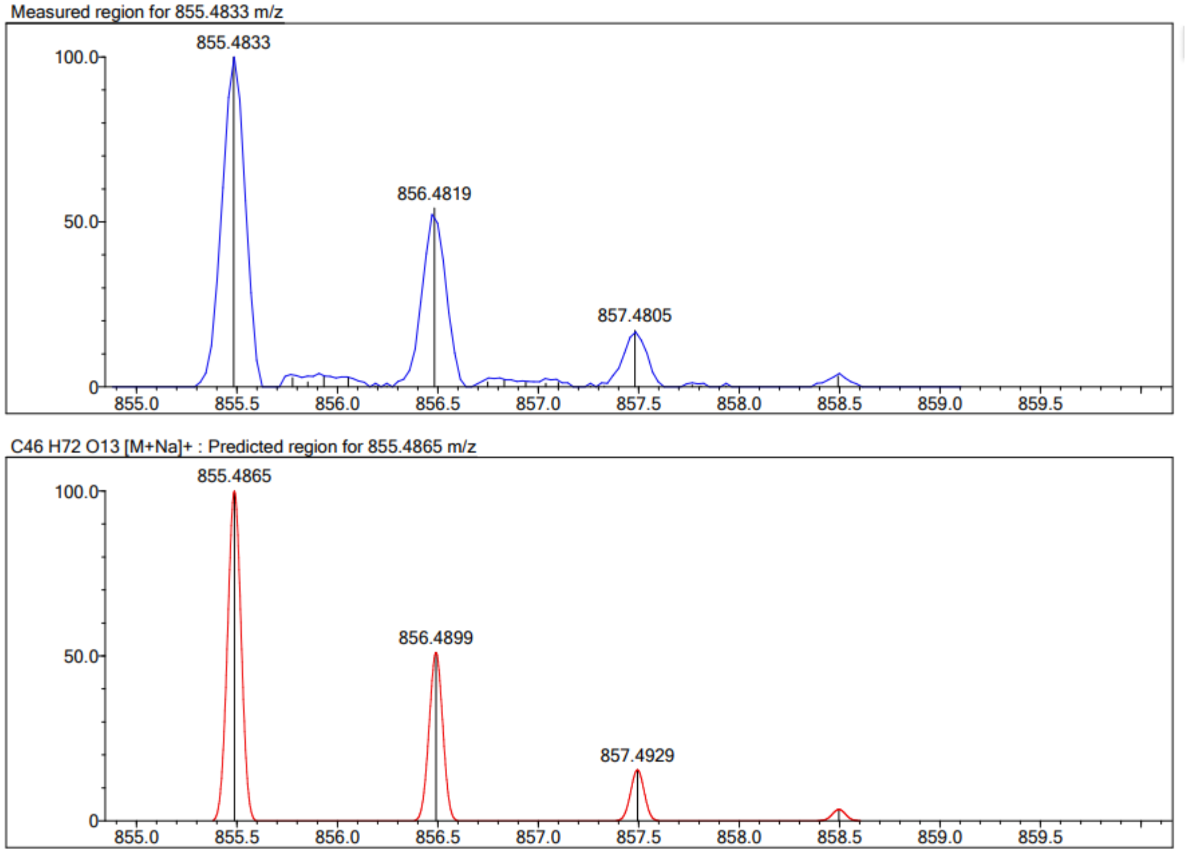

Rank	Score	Formula (M)	Ion	Meas. m/z	Pred. m/z	Df. (mDa)	Df. (ppm)	Iso	DBE
1	84.37	C46 H72 O13	[M+Na]+	855.4833	855.4865	-3.2	-3.74	90.58	11.0

Figure S12. IR (KBr disc) spectrum of **3**.

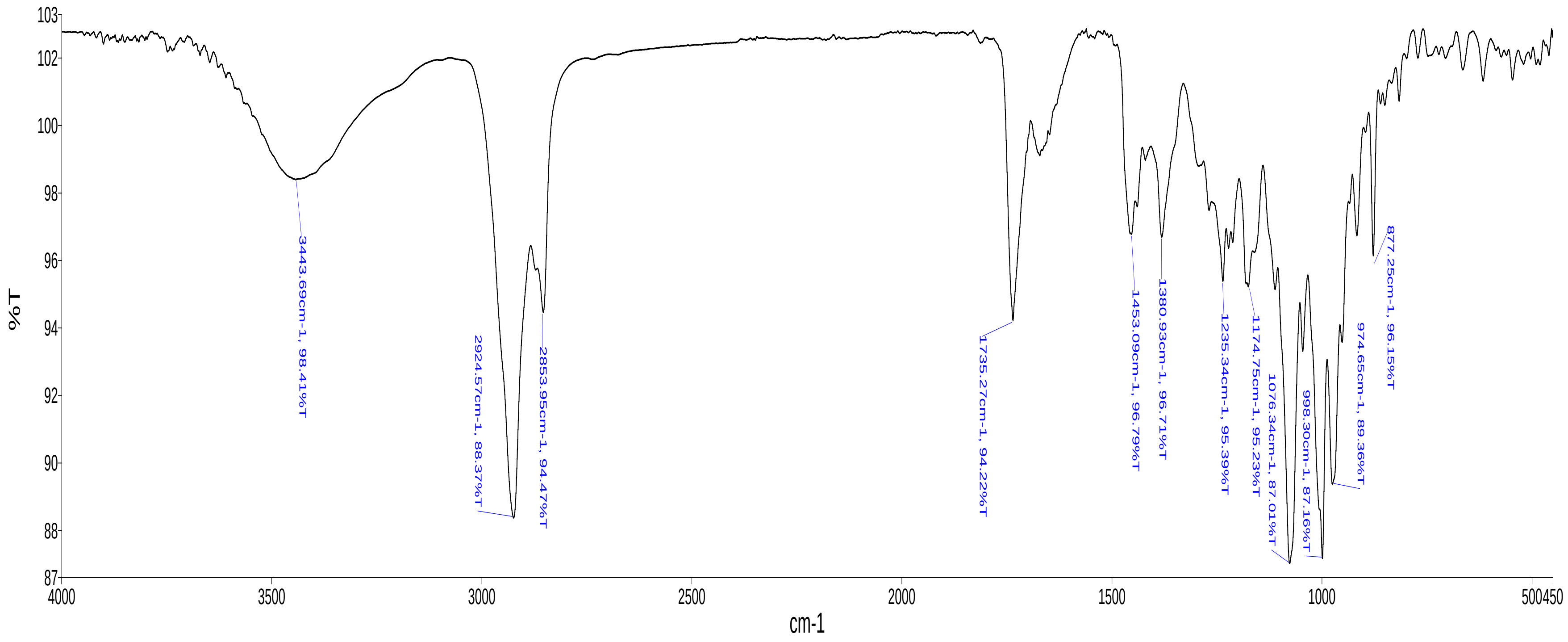

Figure S13. ^{1}H NMR spectrum of **4** in $CDCl_3$.

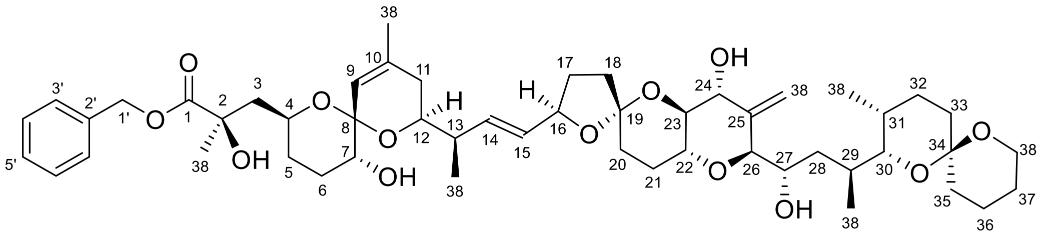

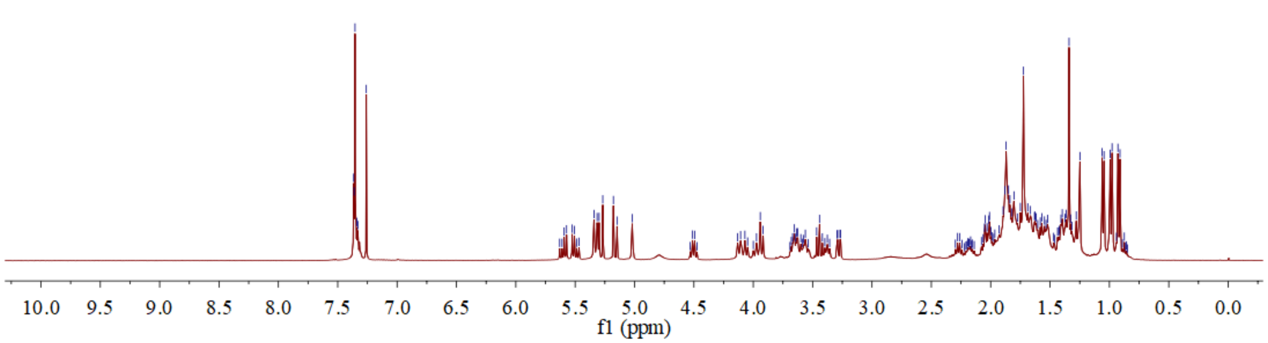

Figure S14. ^{13}C NMR and DEPT 135 spectra of **4** in $CDCl_3$.

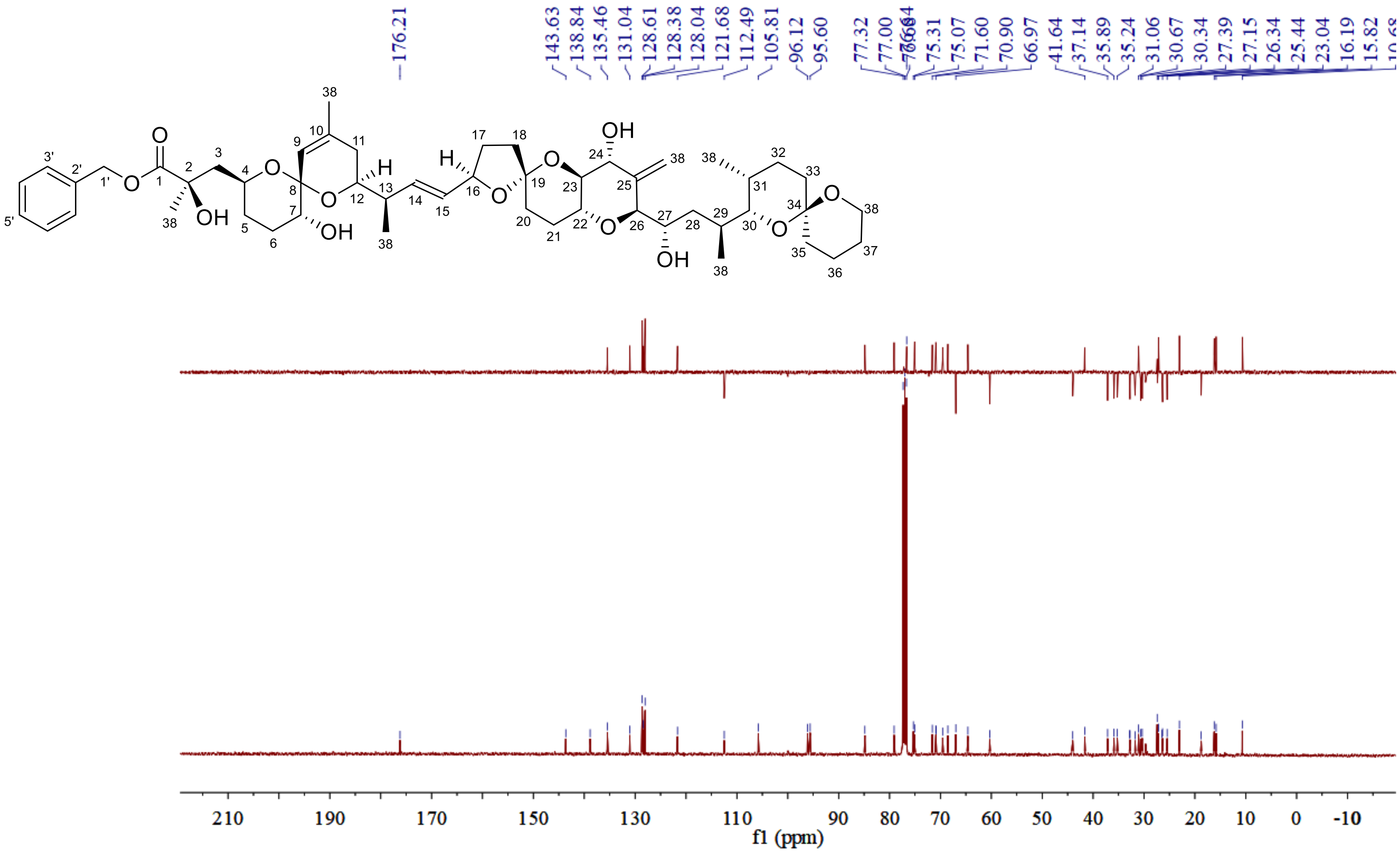

Figure S15. HSQC spectrum of **4** in $CDCl_3$.

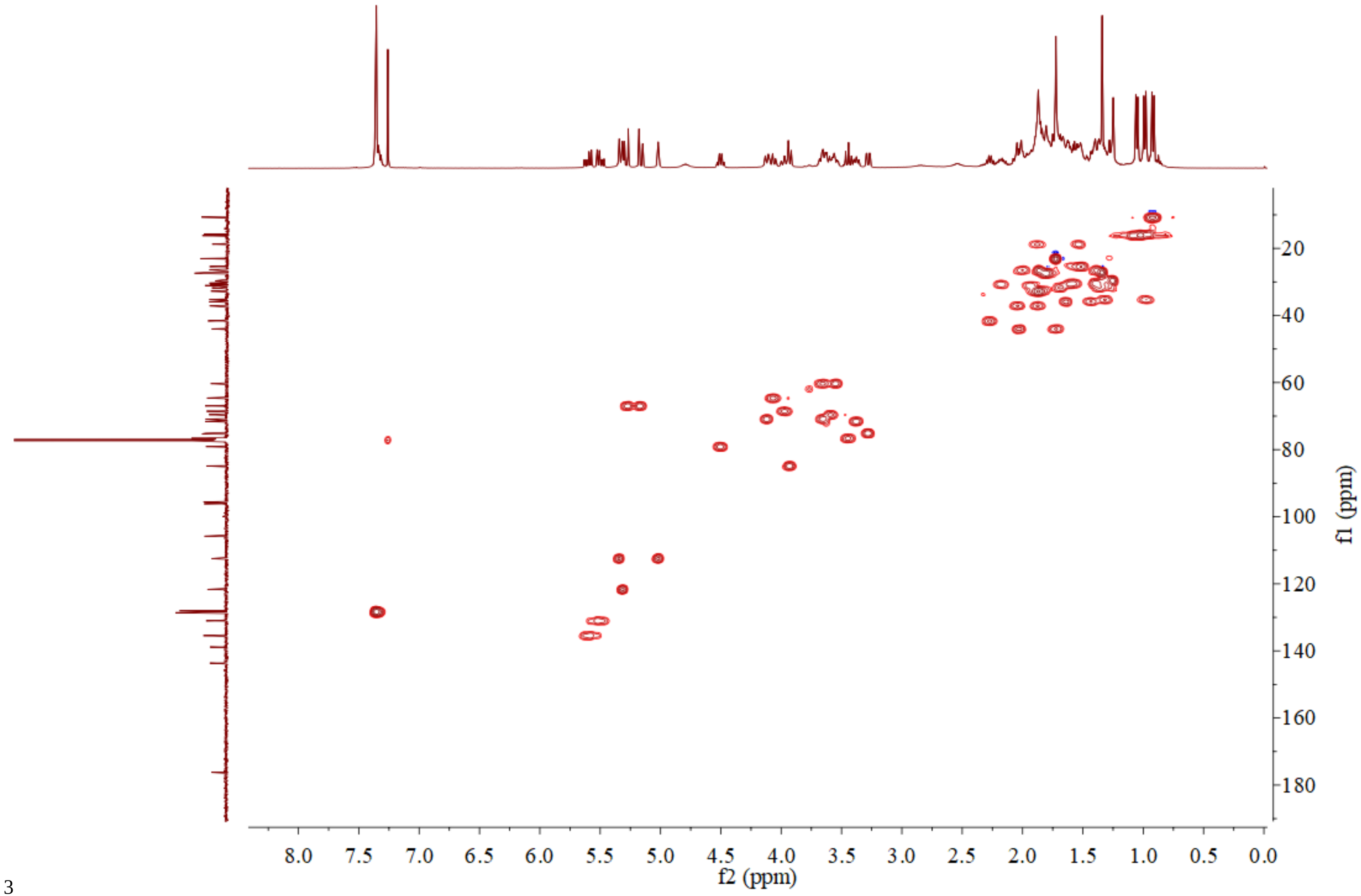

3

Figure S16. HMBC spectrum of **4** in $CDCl_3$.

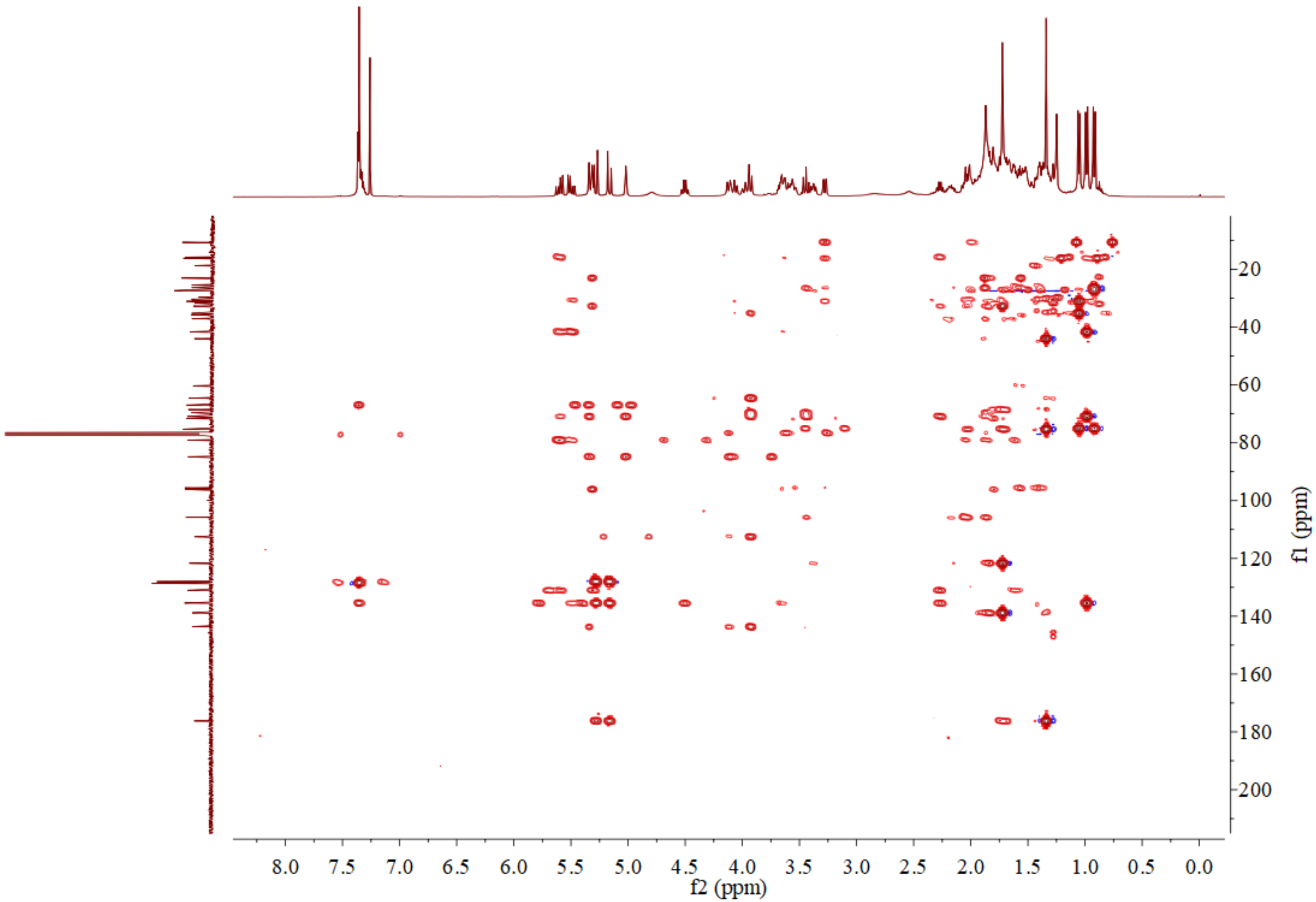

Figure S17. ^1H–^1H COSY spectrum of **4** in $CDCl_3$.

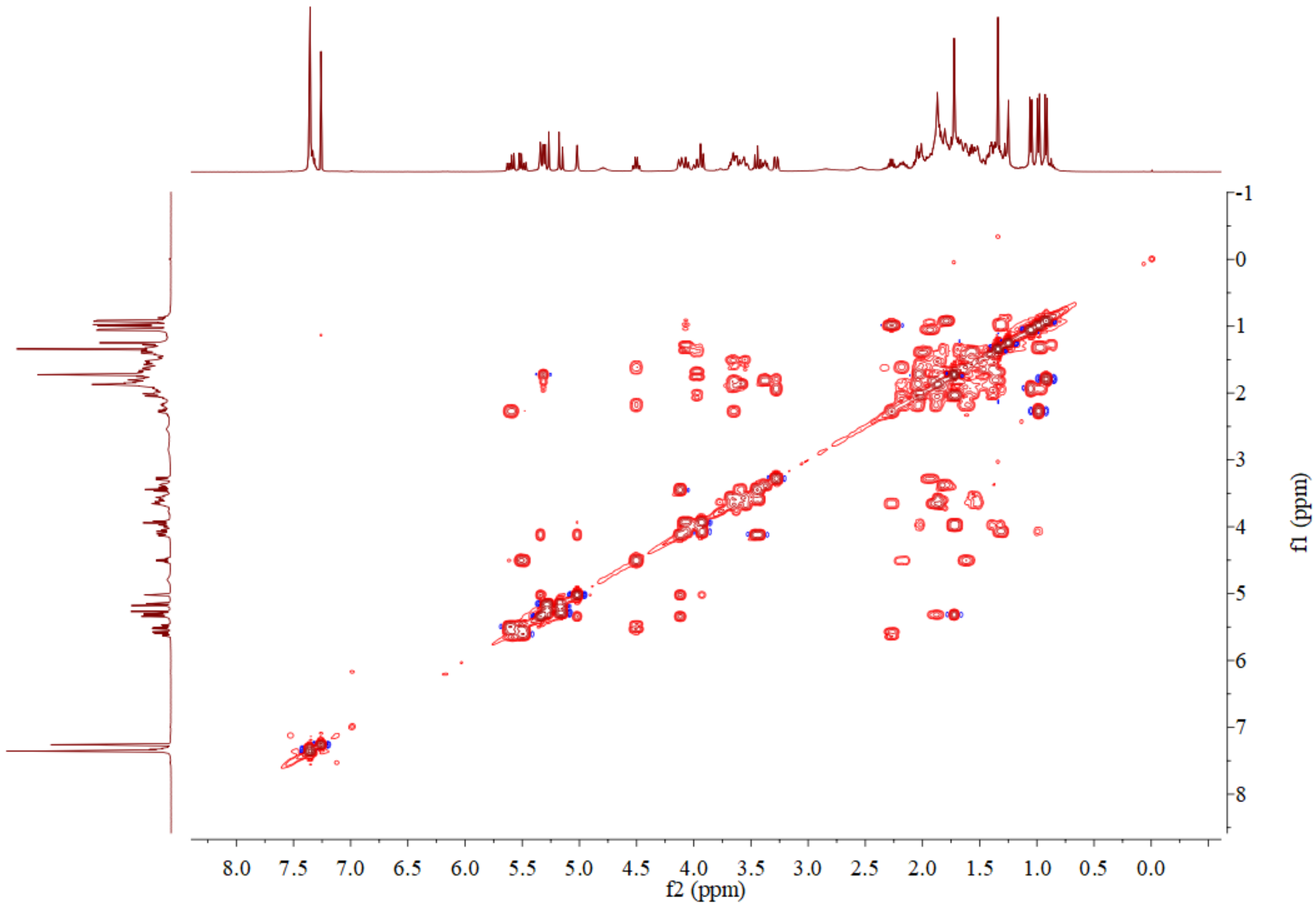

Figure S18. HRESIMS spectrum of **4**.

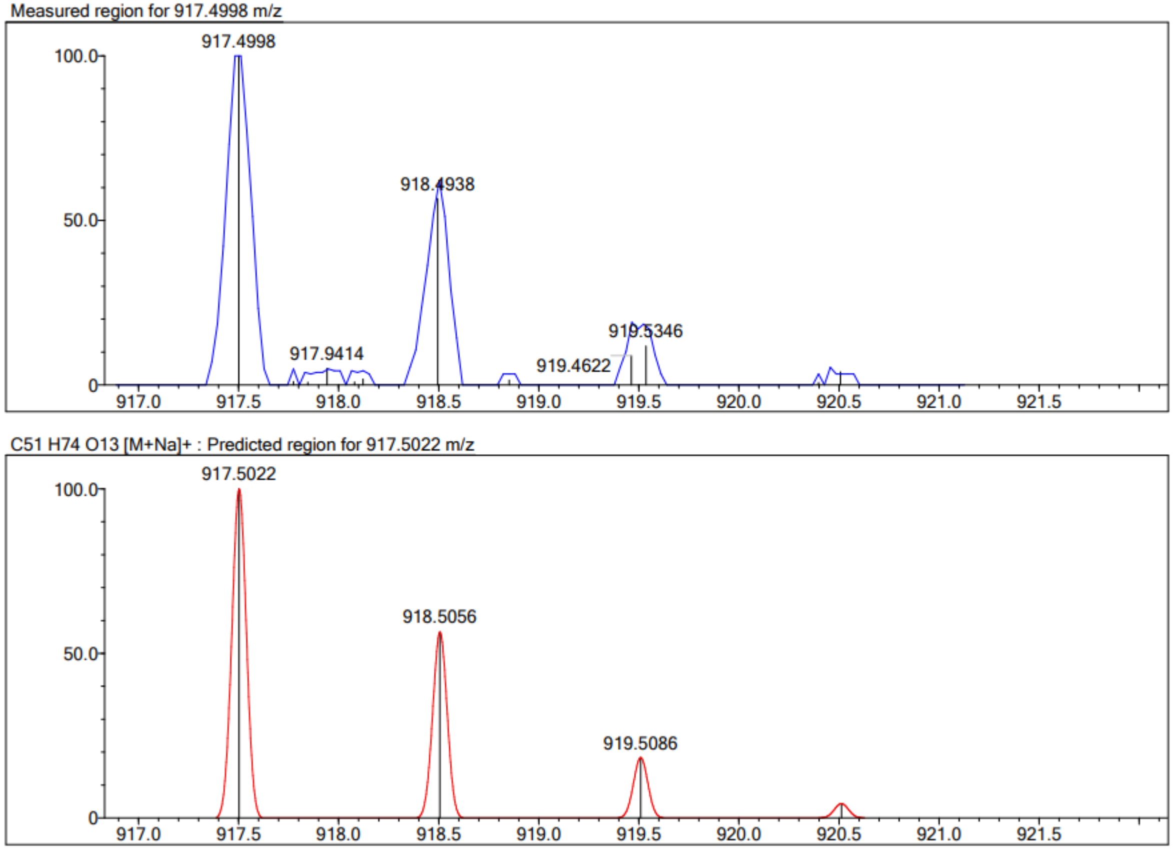

Rank	Score	Formula (M)	Ion	Meas. m/z	Pred. m/z	Df. (mDa)	Df. (ppm)	Iso	DBE
1	89.11	C51 H74 O13	[M+Na]+	917.4998	917.5022	-2.4	-2.62	92.87	15.0

Figure S19. IR (KBr disc) spectrum of **4**.

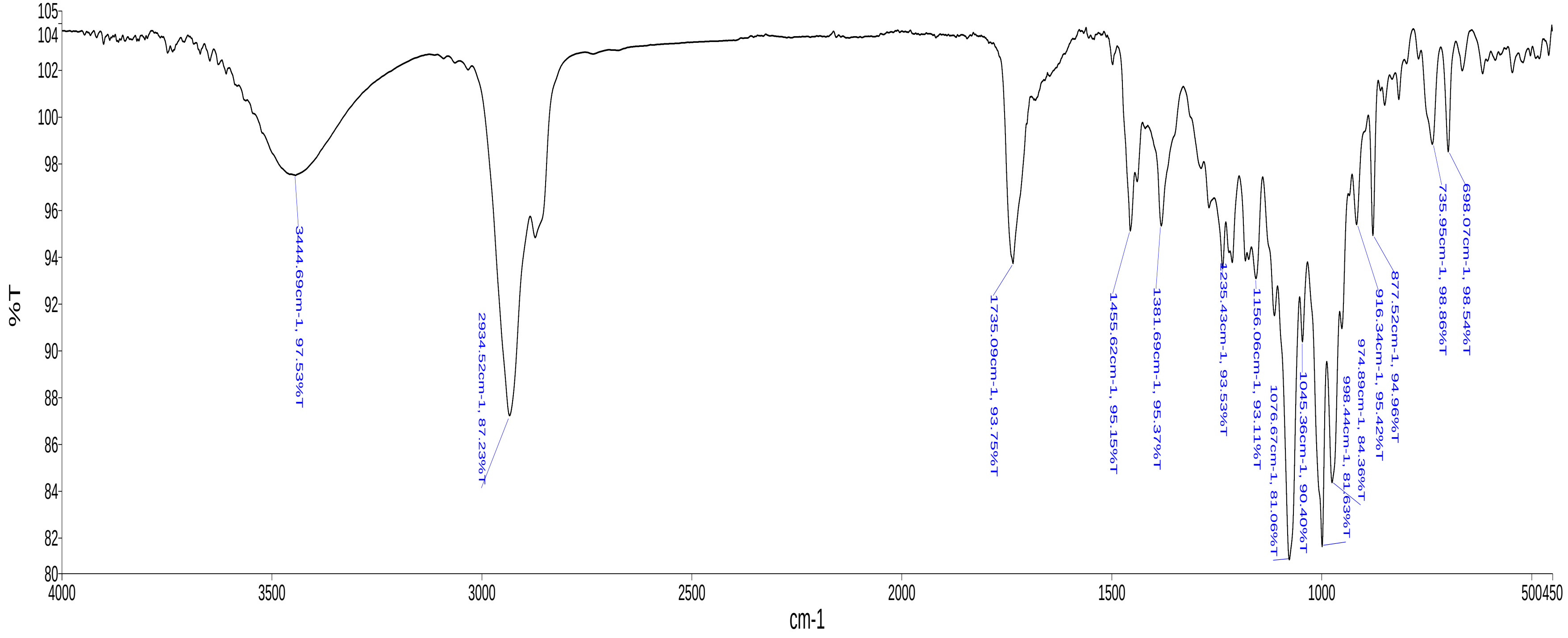

Figure S20. ^{1}H NMR spectrum of **5** in $CDCl_3$.

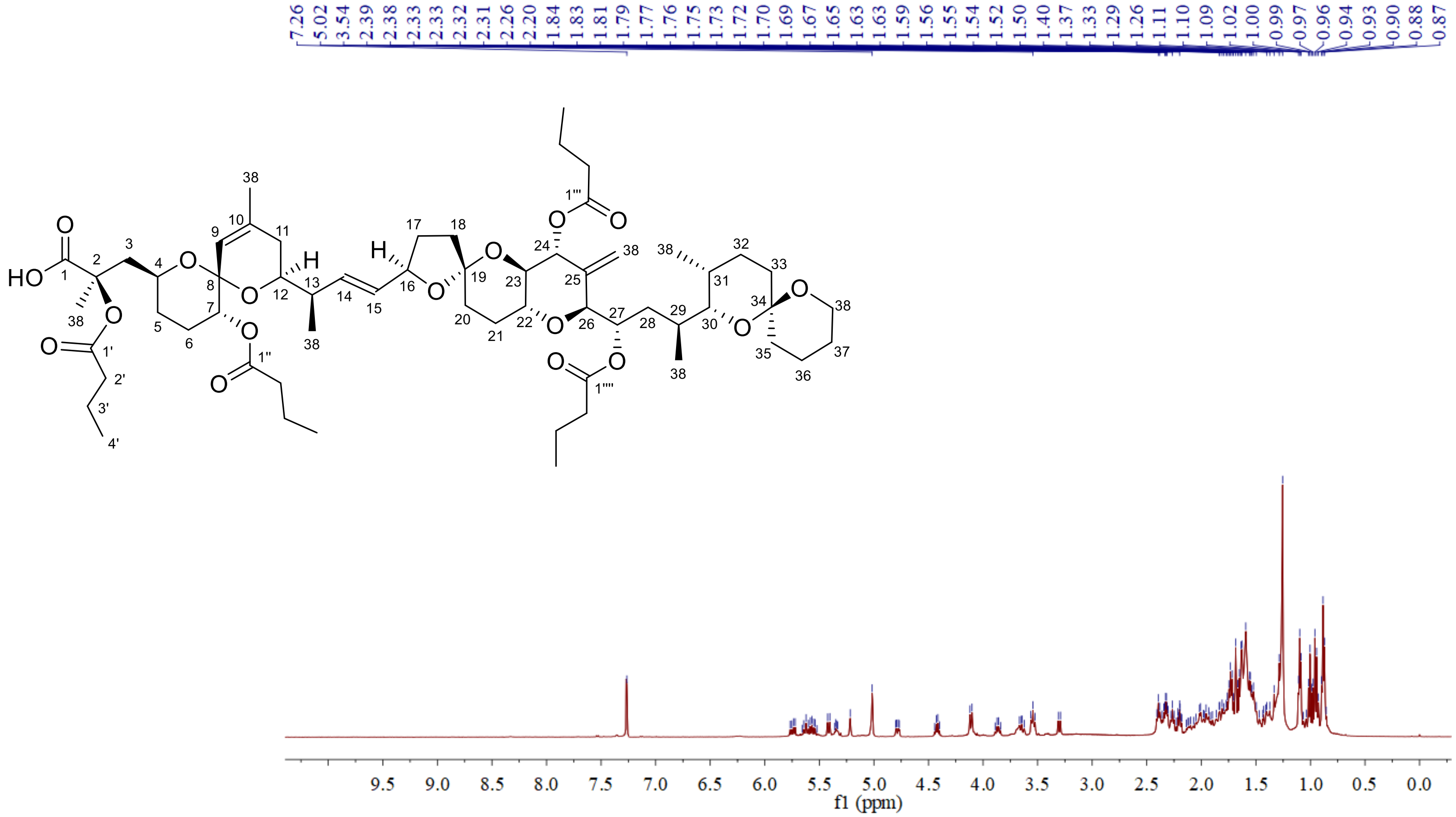

Figure S21. ^{13}C NMR and DEPT 135 spectra of **5** in $CDCl_3$.

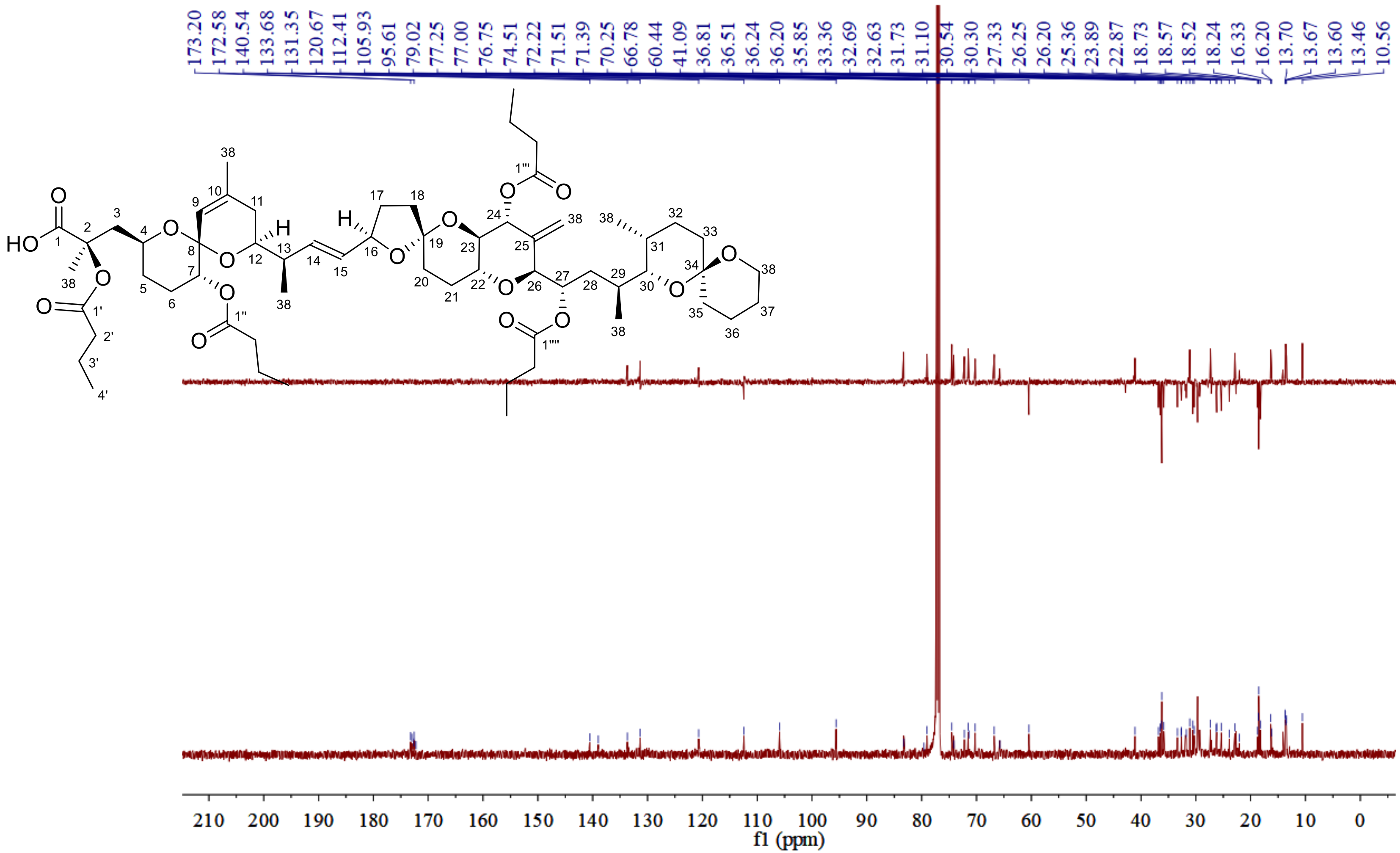

Figure S22. HSQC spectrum of **5** in $CDCl_3$.

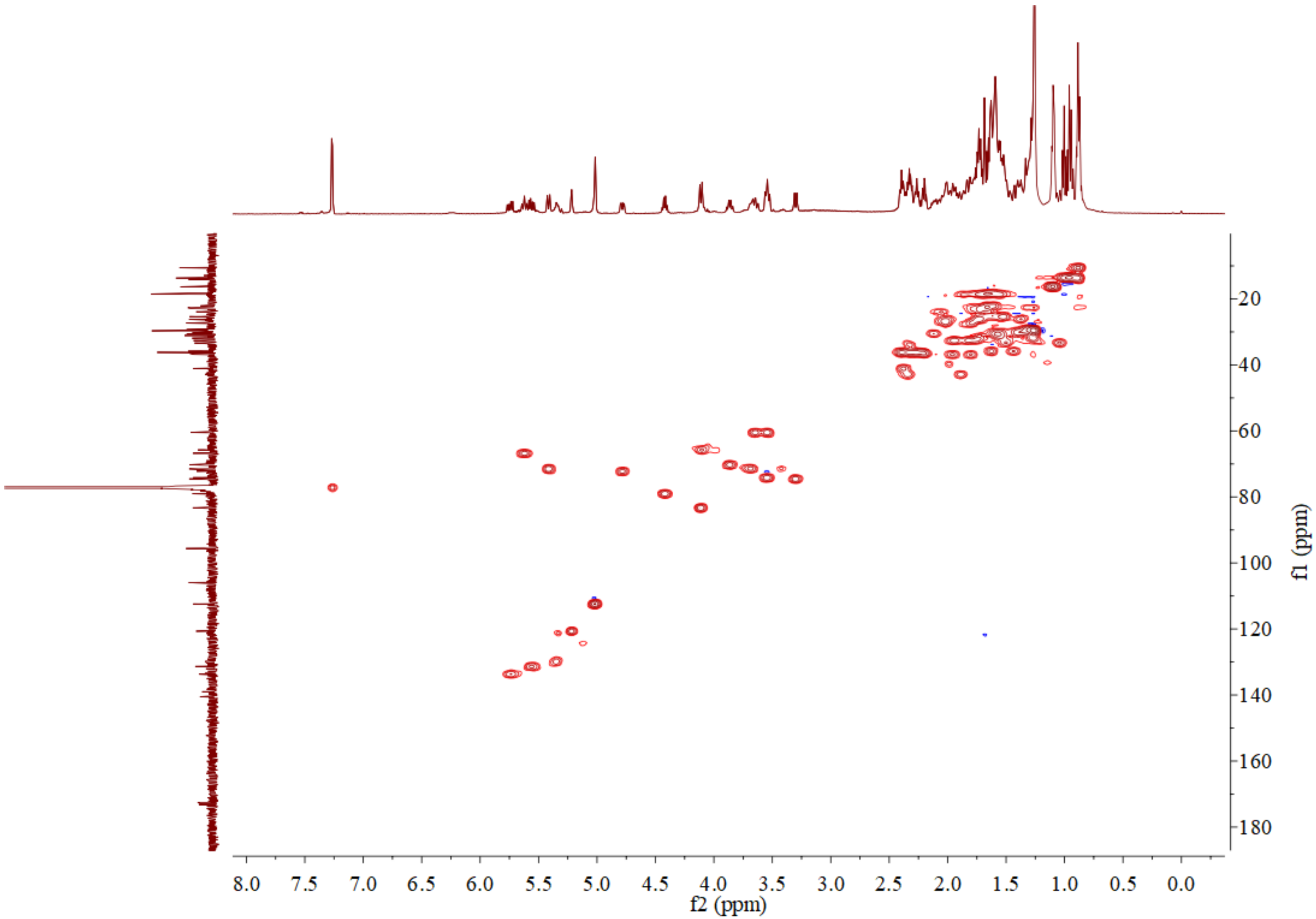

Figure S23. HMBC spectrum of **5** in $CDCl_3$.

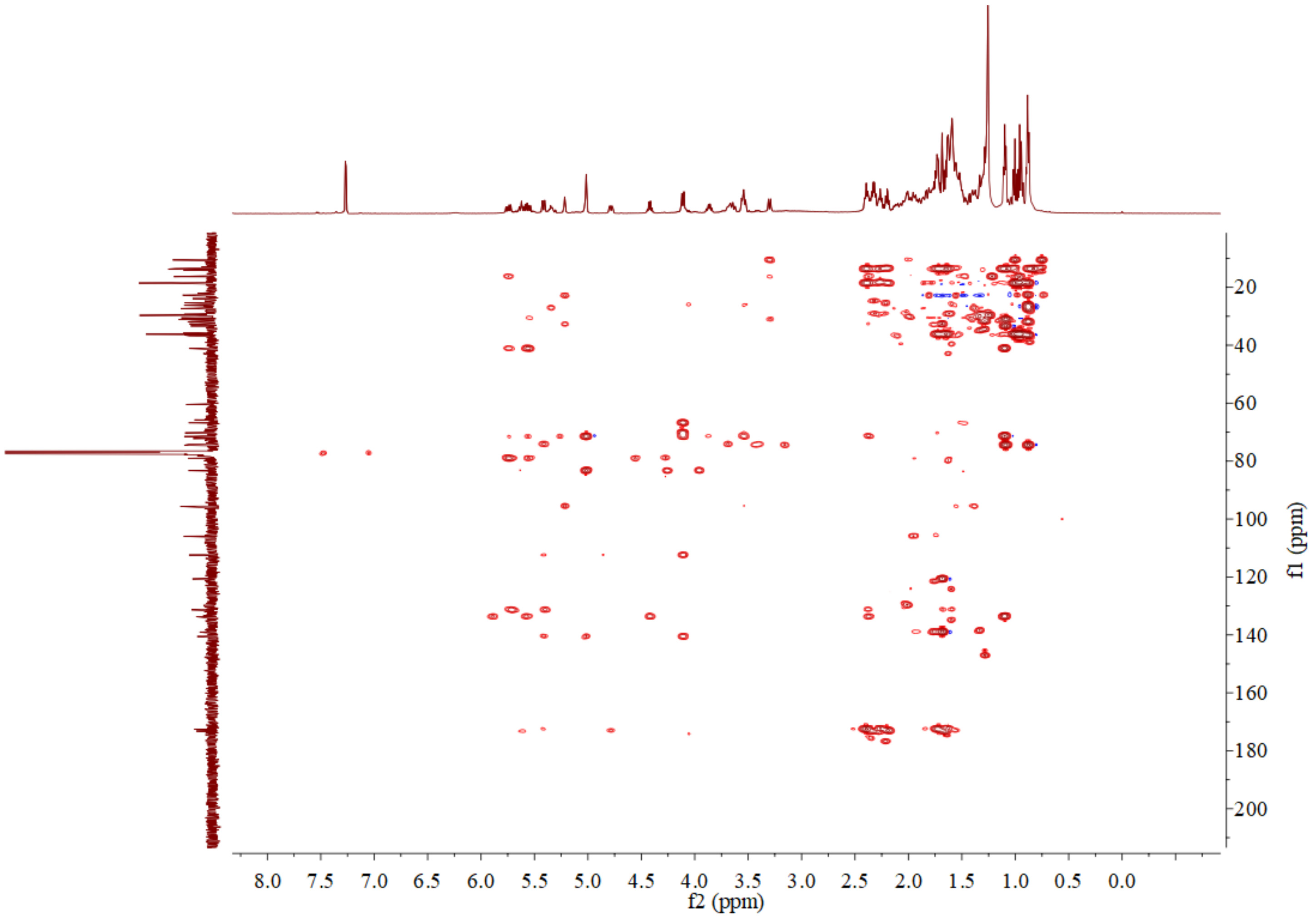

Figure S24. ^1H–^1H COSY spectrum of **5** in $CDCl_3$.

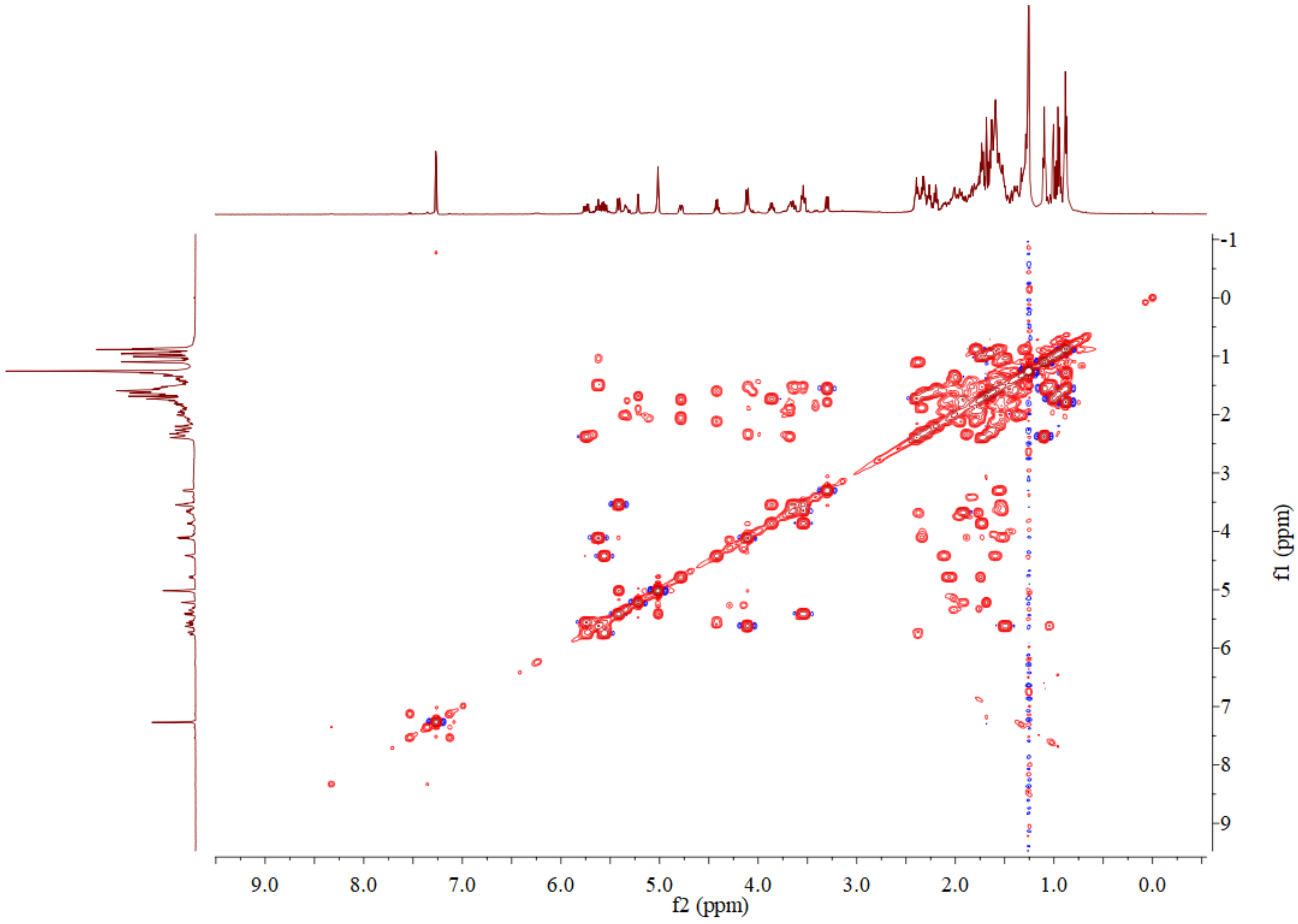

Figure S25. HRESIMS spectrum of **5**.

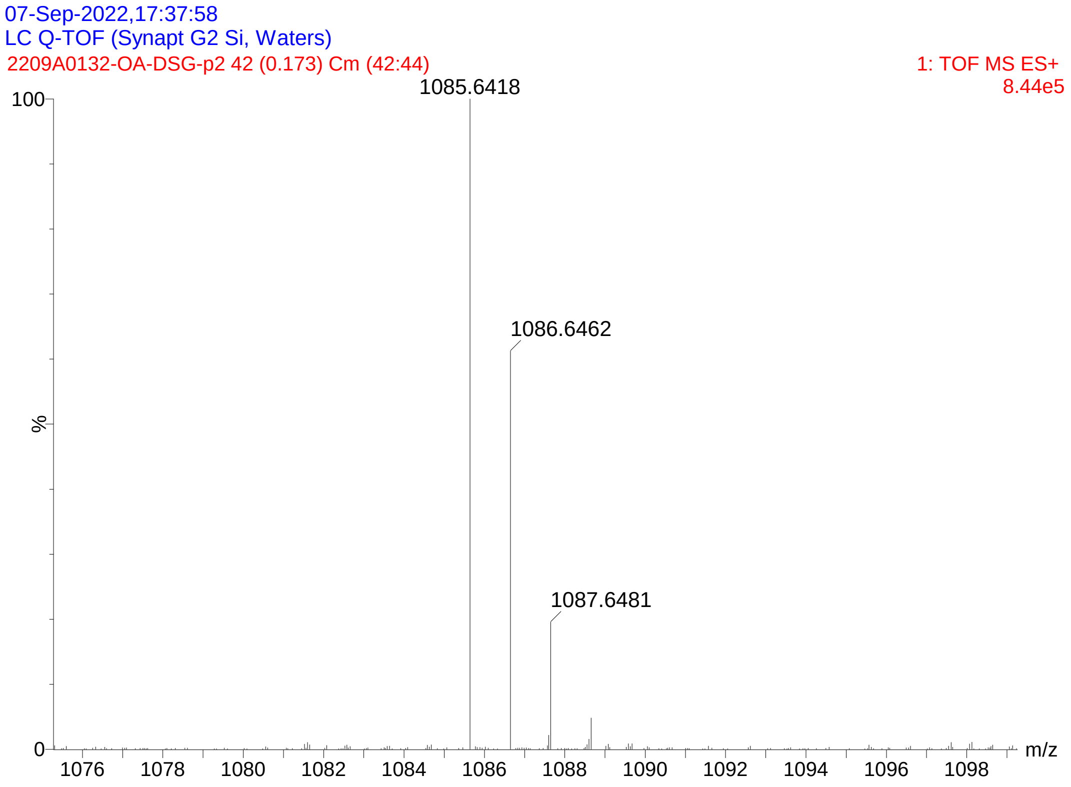

Tolerance = 10.0 PPM / DBE: min = -1.5, max = 50.0

Elements Used:

C: 0-80 H: 0-120 O: 0-20

Mass	Calc. Mass	mDa	PPM	DBE	Formula
1085.6418	1085.6413	0.5	0.5	14.5	$C_{60}H_{93}O_{17}$

Figure S26. IR (KBr disc) spectrum of **5**.

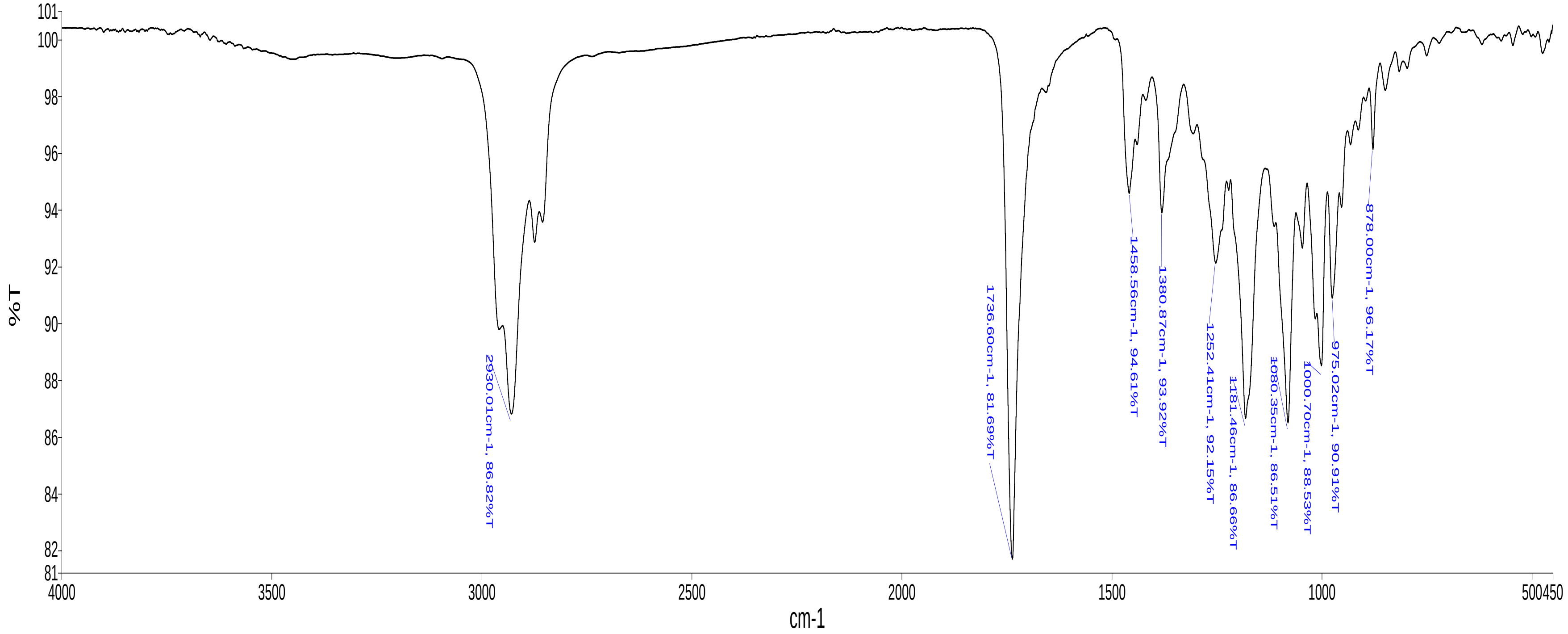

Figure S27. ^{1}H NMR spectrum of **6** in $CDCl_3$.

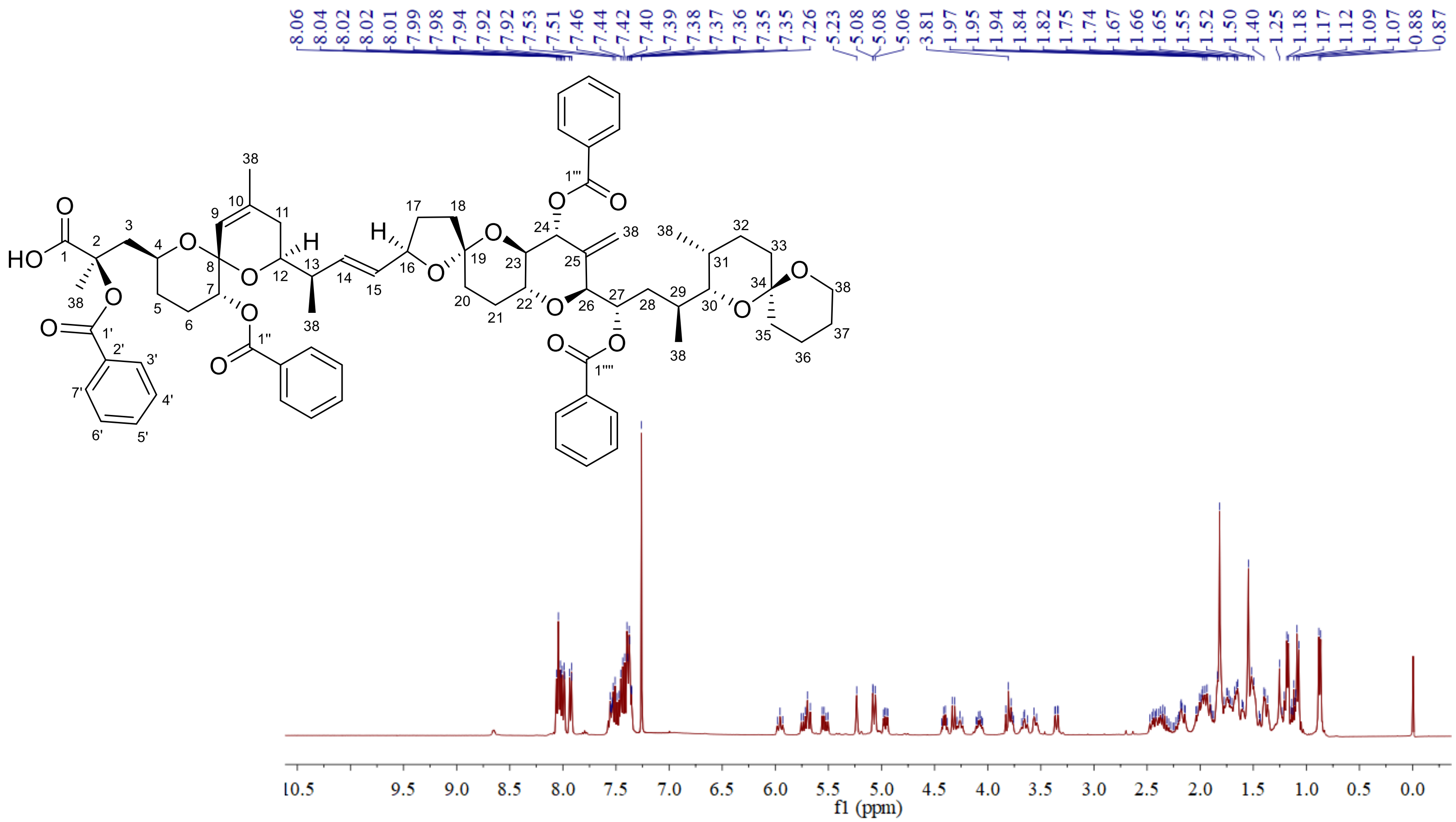

Figure S28. ^{13}C NMR and DEPT 135 spectra of **6** in $CDCl_3$.

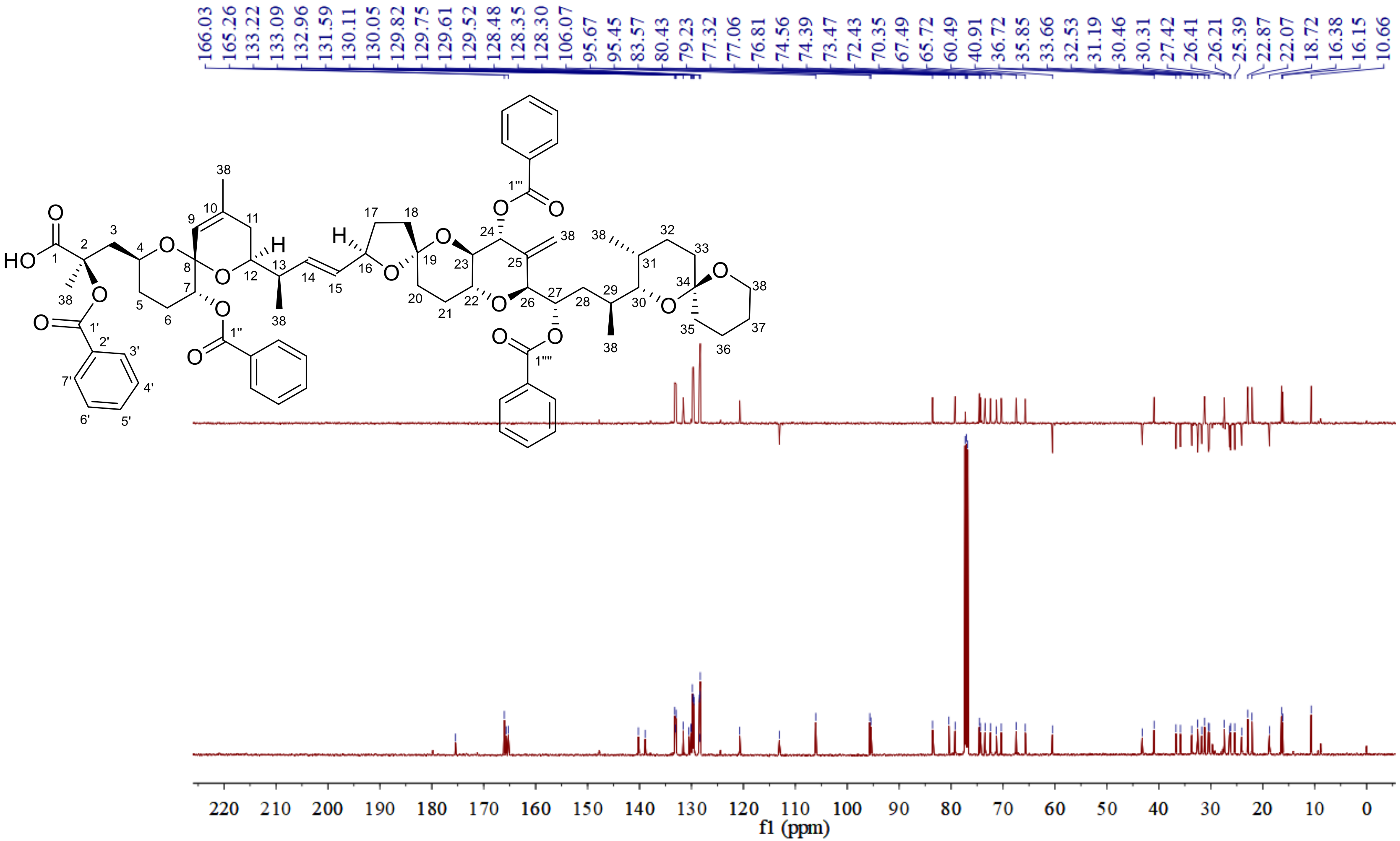

Figure S29. HSQC spectrum of **6** in $CDCl_3$.

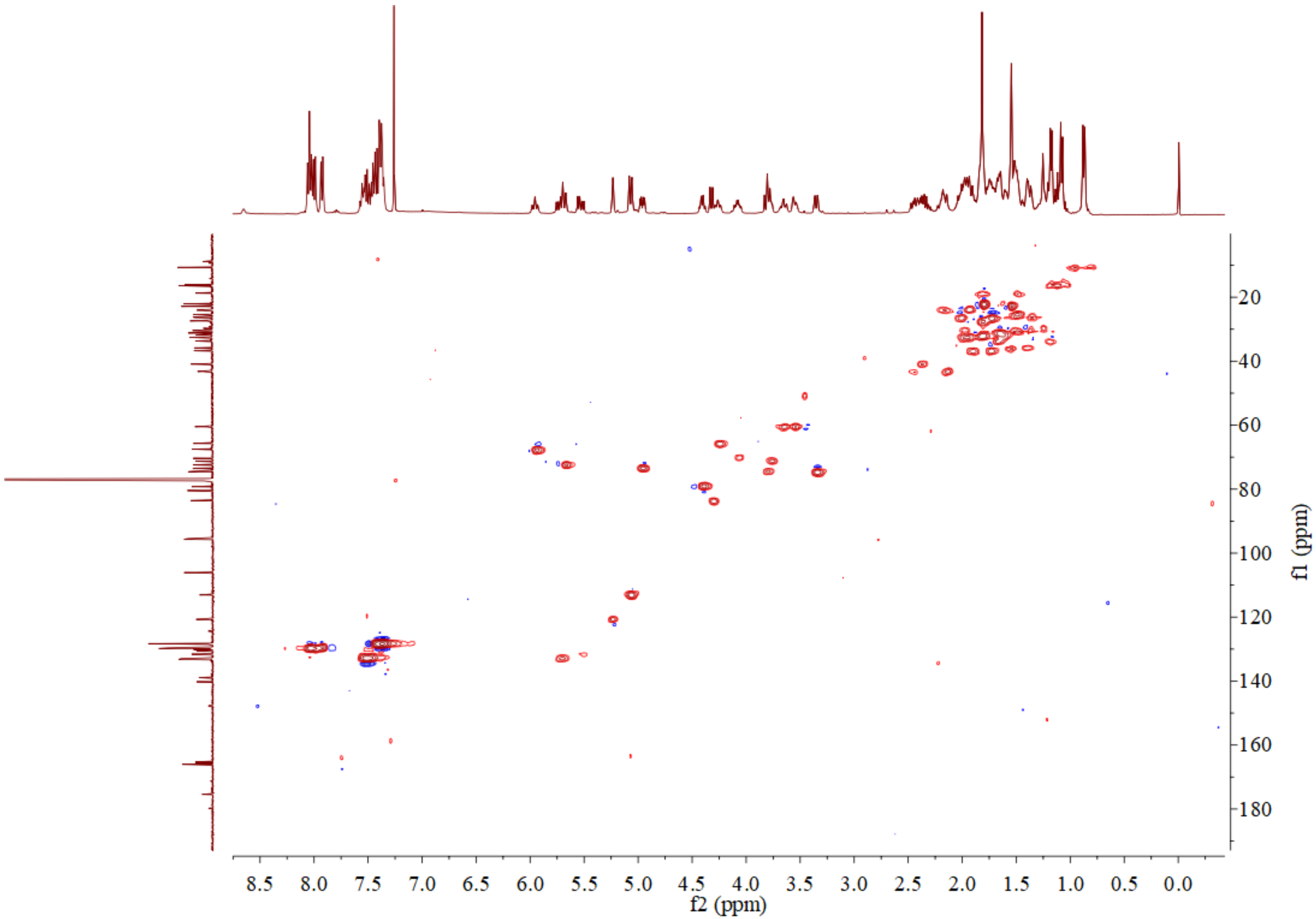

Figure S30. HMBC spectrum of **6** in $CDCl_3$.

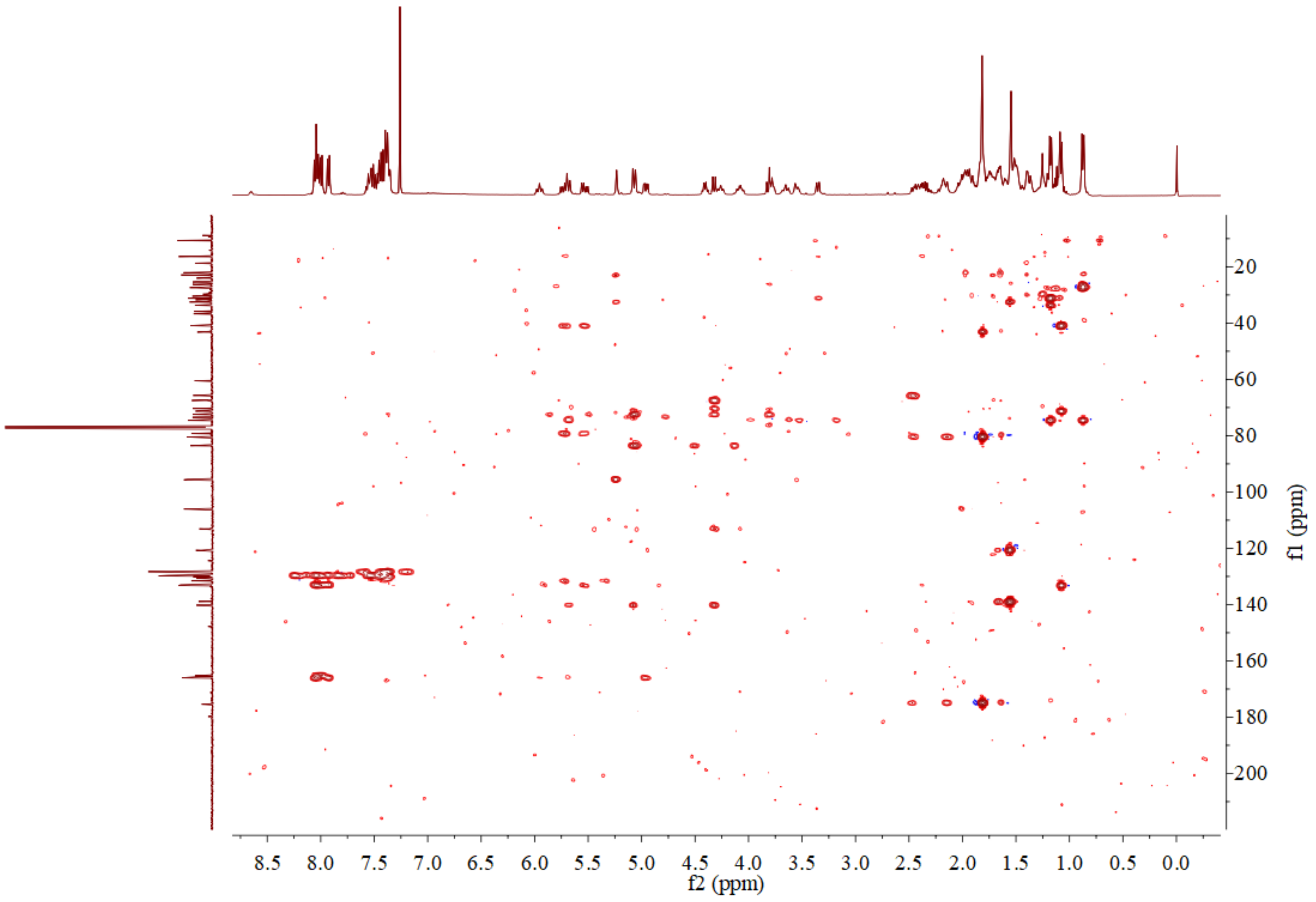

Figure S31. ^1H–^1H COSY spectrum of **6** in $CDCl_3$.

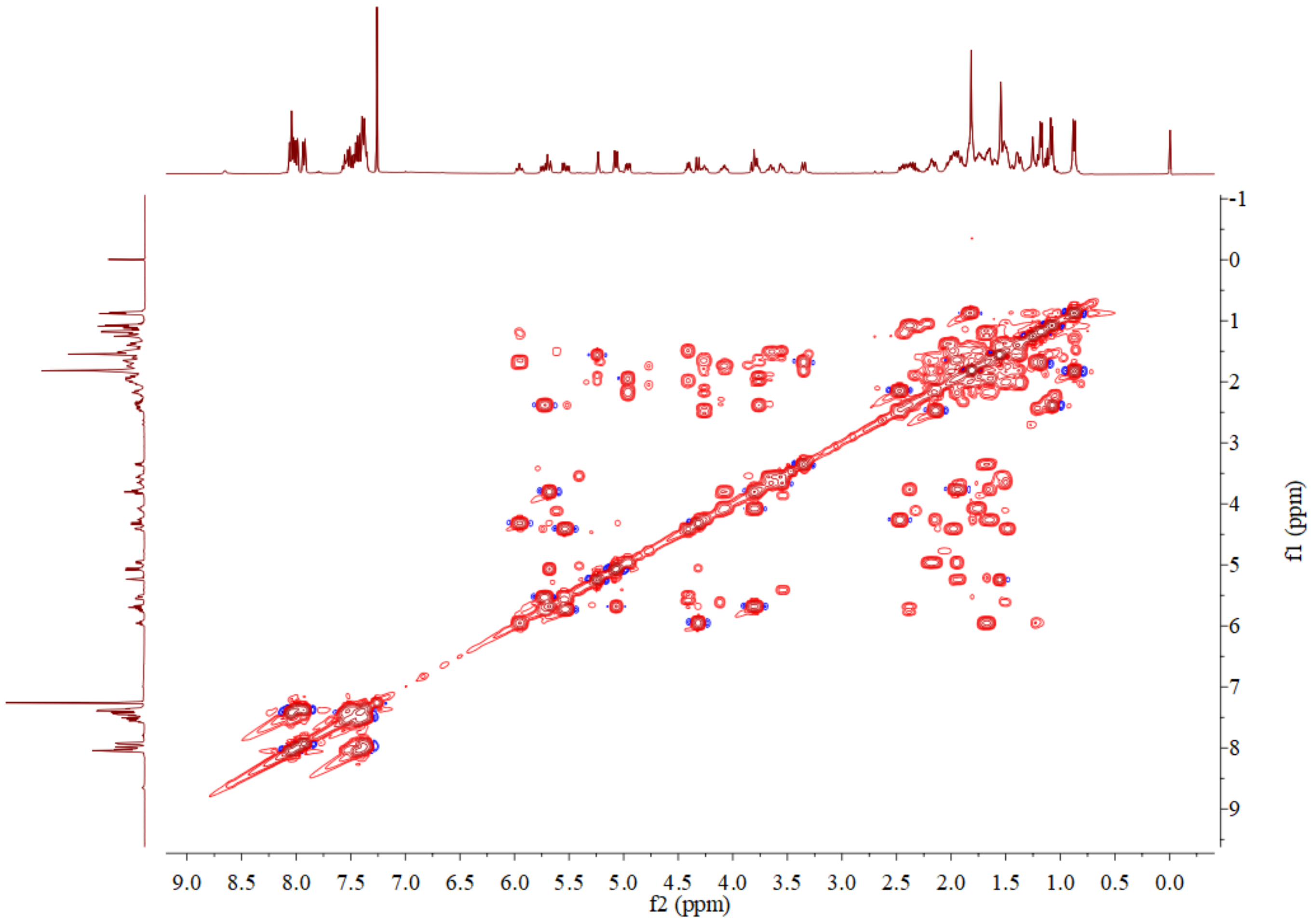

Figure S32. HRESIMS spectrum of **6**.

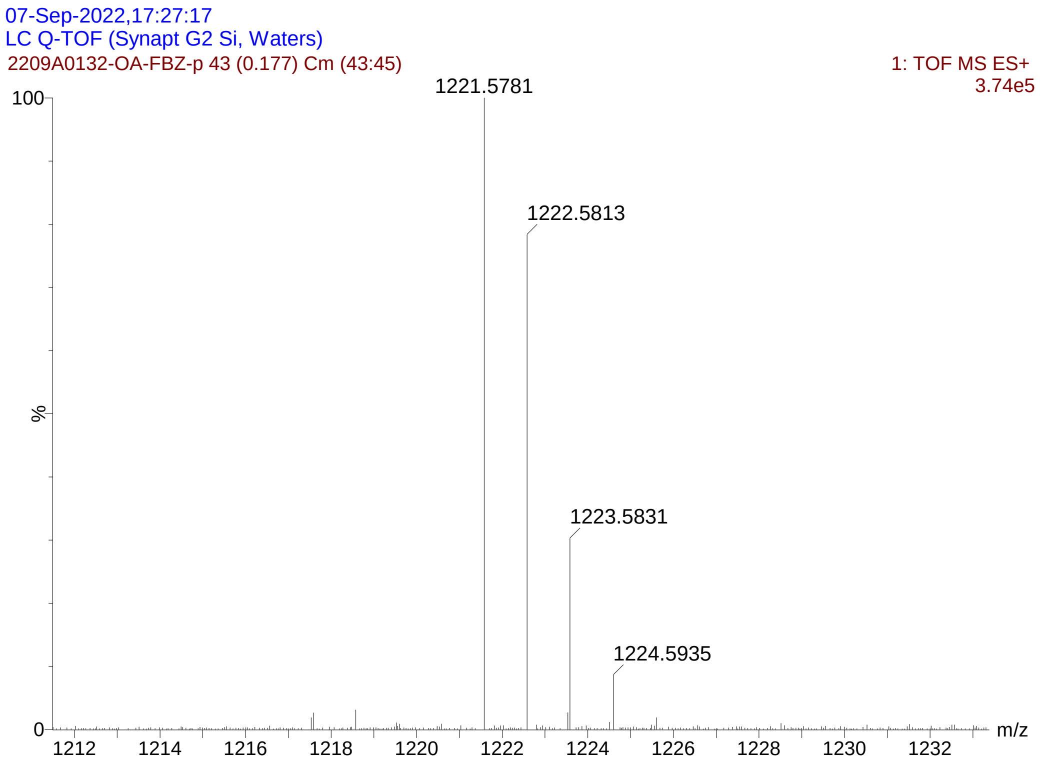

Tolerance = 10.0 PPM / DBE: min = -1.5, max = 50.0

Elements Used:

C: 0-80 H: 0-120 O: 0-20

Mass	Calc. Mass	mDa	PPM	DBE	Formula
1221.5781	1221.5787	-0.6	-0.5	30.5	$C_{72}H_{85}O_{17}$

Figure S33. IR (KBr disc) spectrum of **6**.

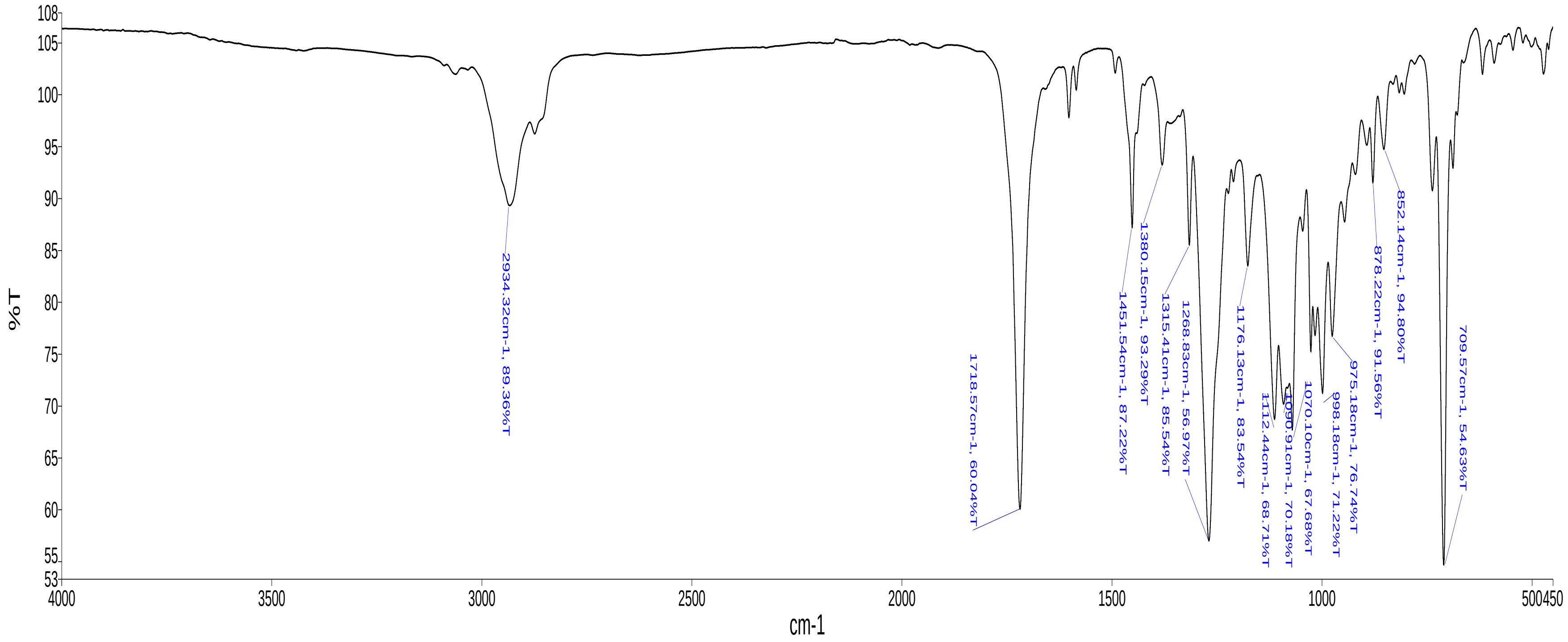

Figure S34. ^{1}H NMR spectrum of **7** in $CDCl_3$.

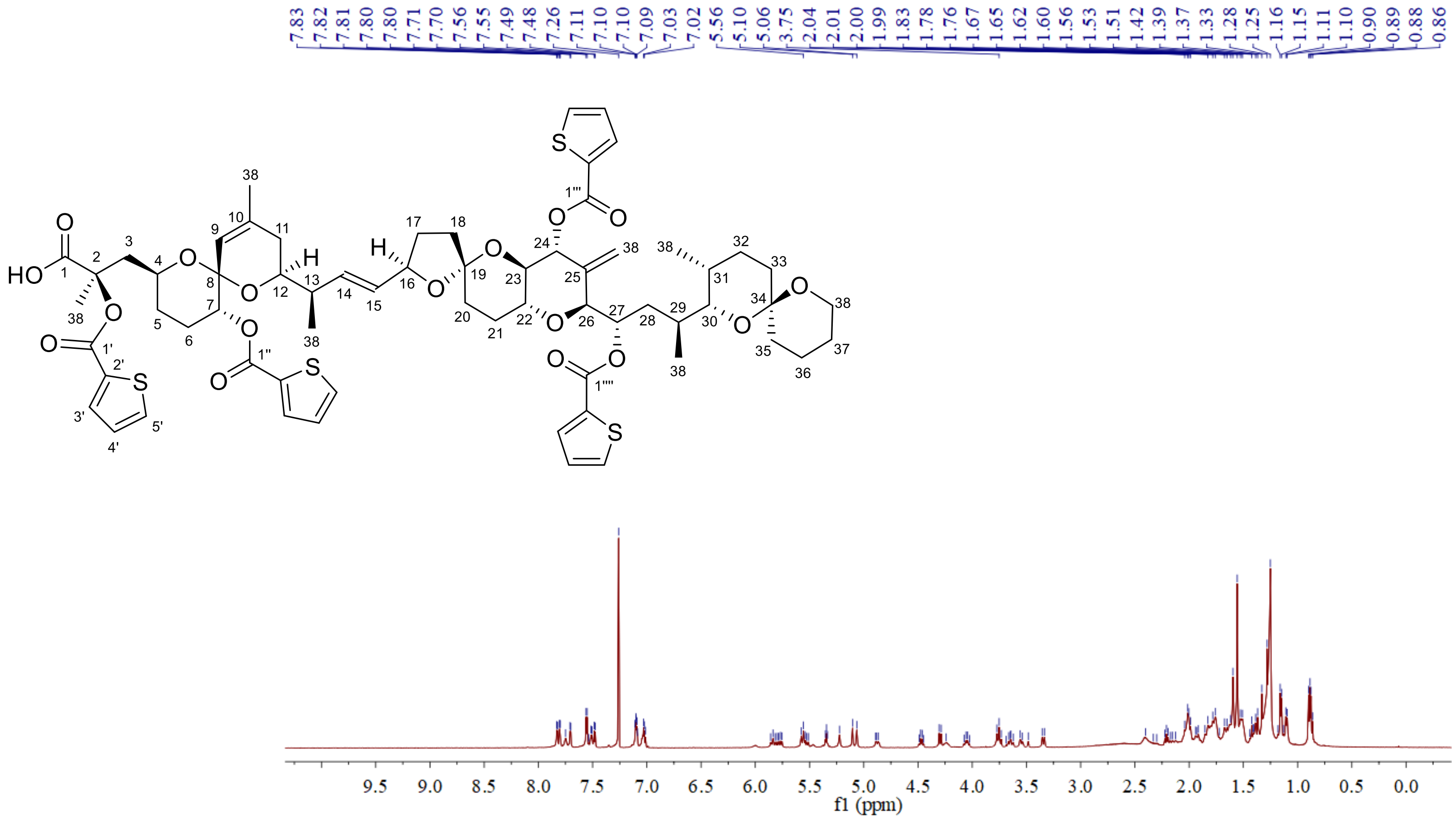

Figure S35. ^{13}C NMR and DEPT 135 spectra of **7** in $CDCl_3$.

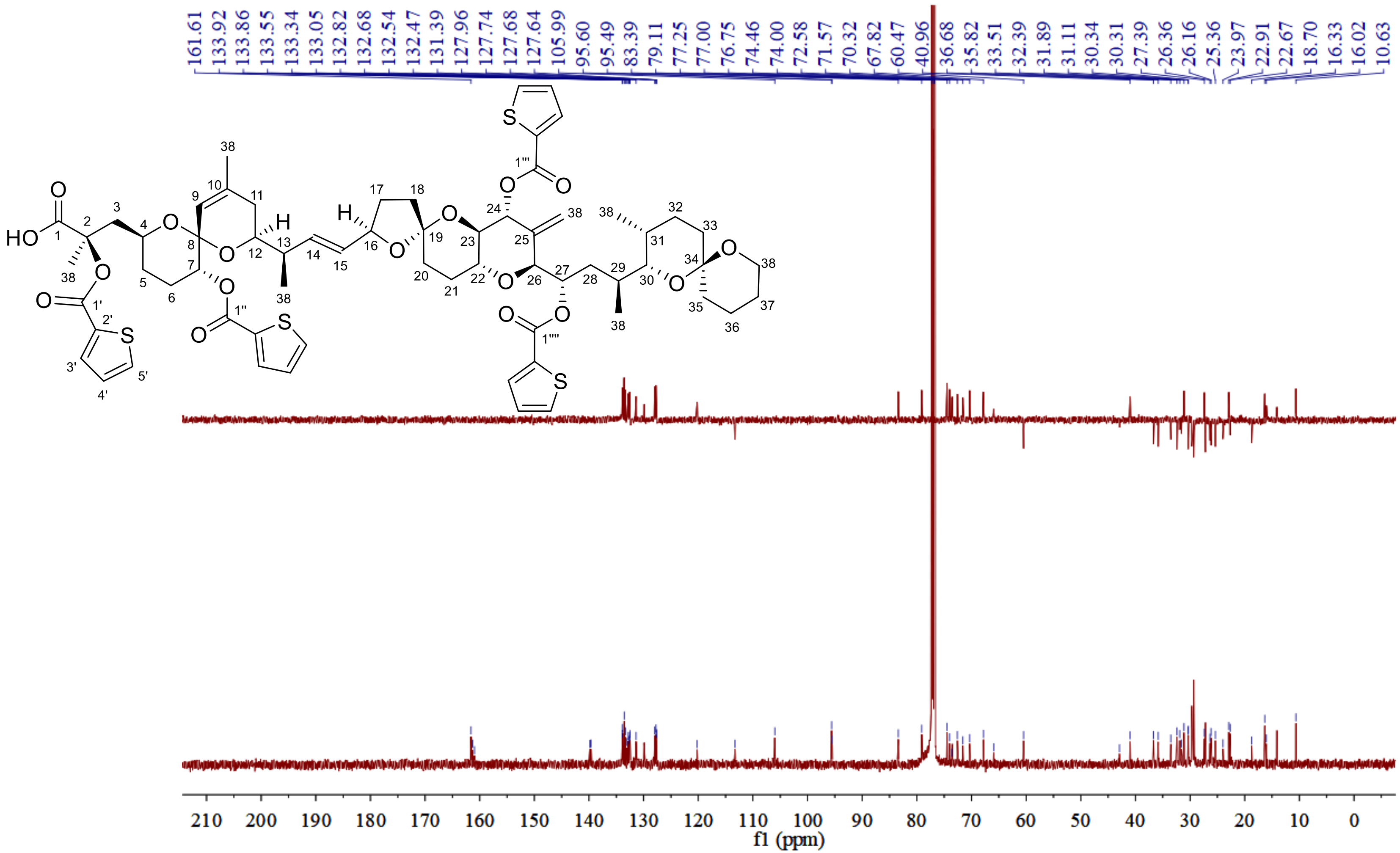

Figure S36. HMBC spectrum of **7** in $CDCl_3$.

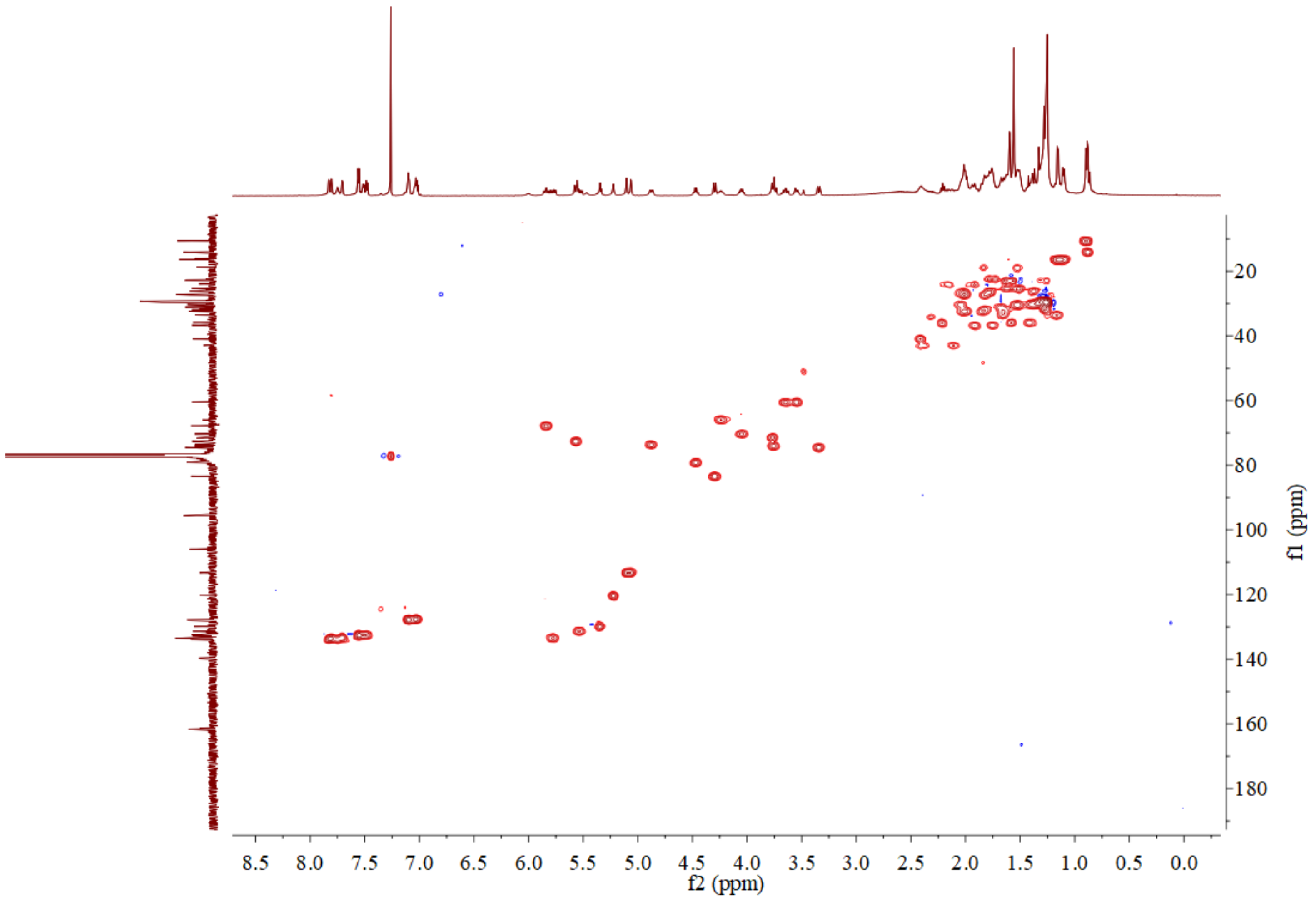

Figure S37. HMBC spectrum of **7** in $CDCl_3$.

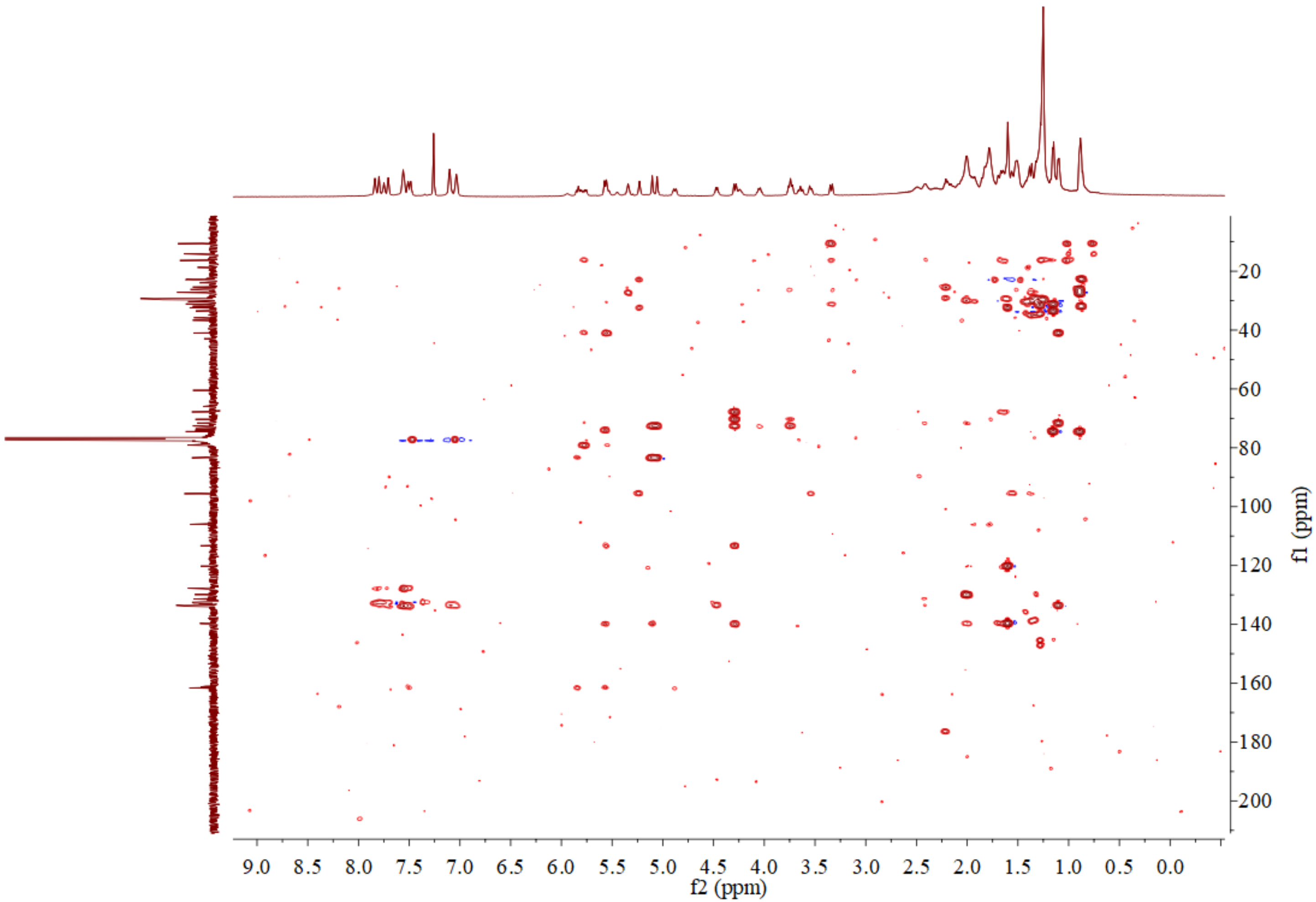

Figure S38. ^{1}H–^{1}H COSY spectrum of **7** in $CDCl_3$.

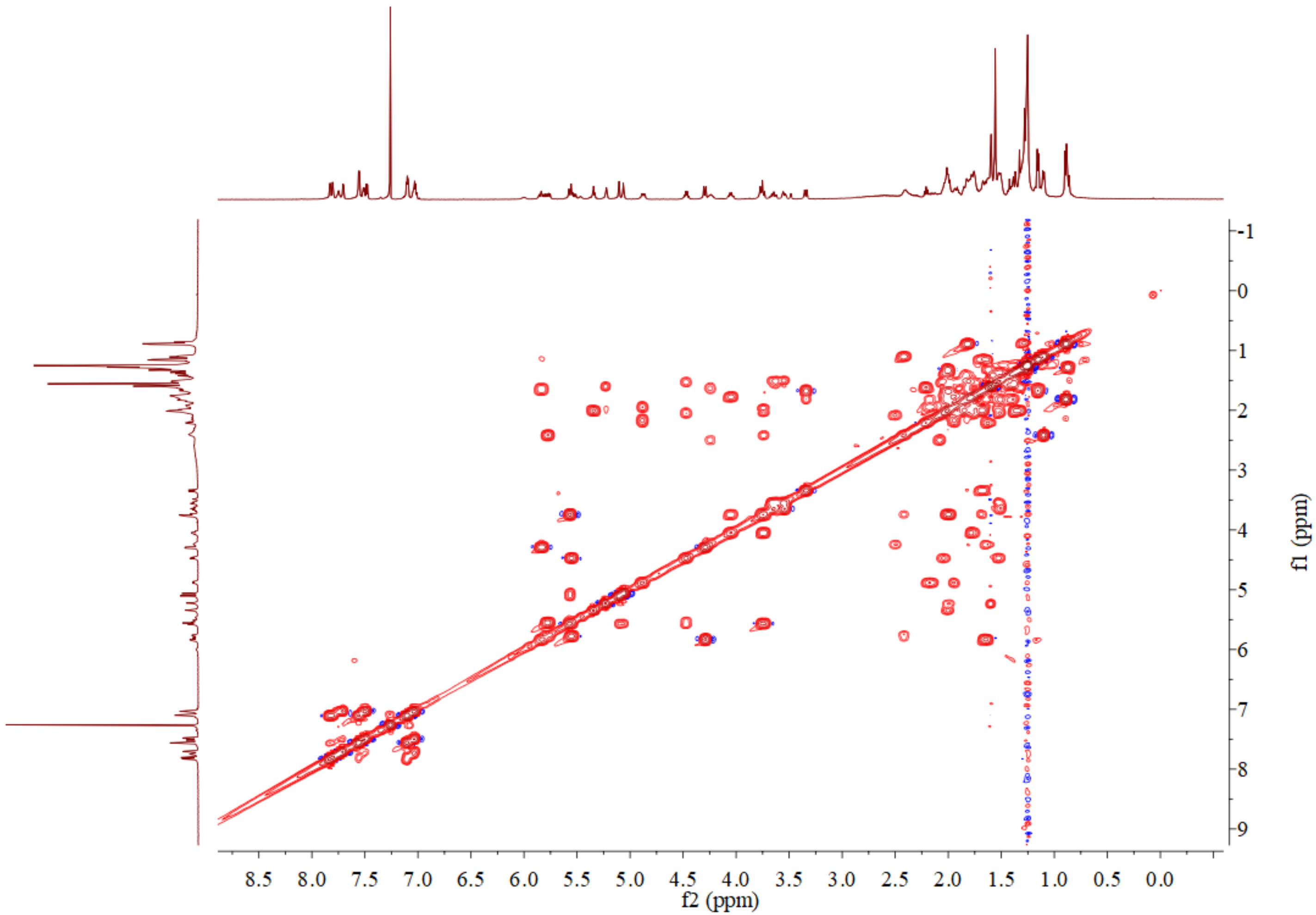

Figure S39. HRESIMS spectrum of **7**.

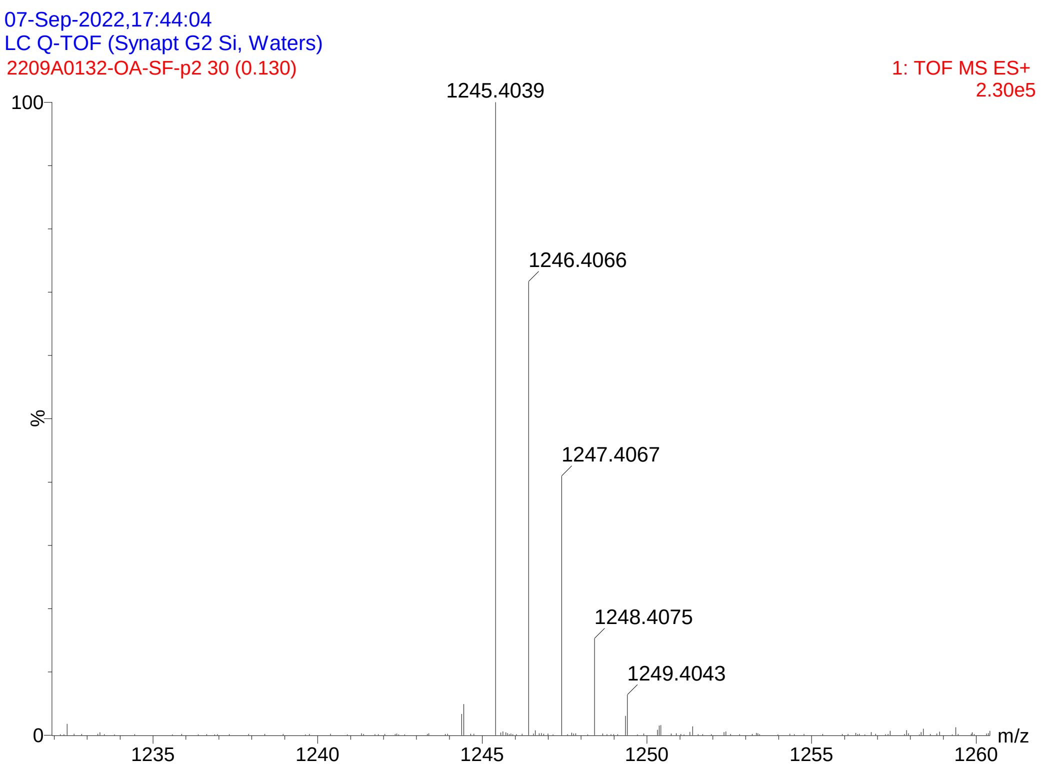

Tolerance = 10.0 PPM / DBE: min = -1.5, max = 50.0

Elements Used:

C: 0-80 H: 0-120 O: 0-20 S: 4-4

Mass	Calc. Mass	mDa	PPM	DBE	Formula
1245.4039	1245.4044	-0.5	-0.4	26.5	$C_{64}H_{77}O_{17}S_4$

Figure S40. IR (KBr disc) spectrum of **7**.

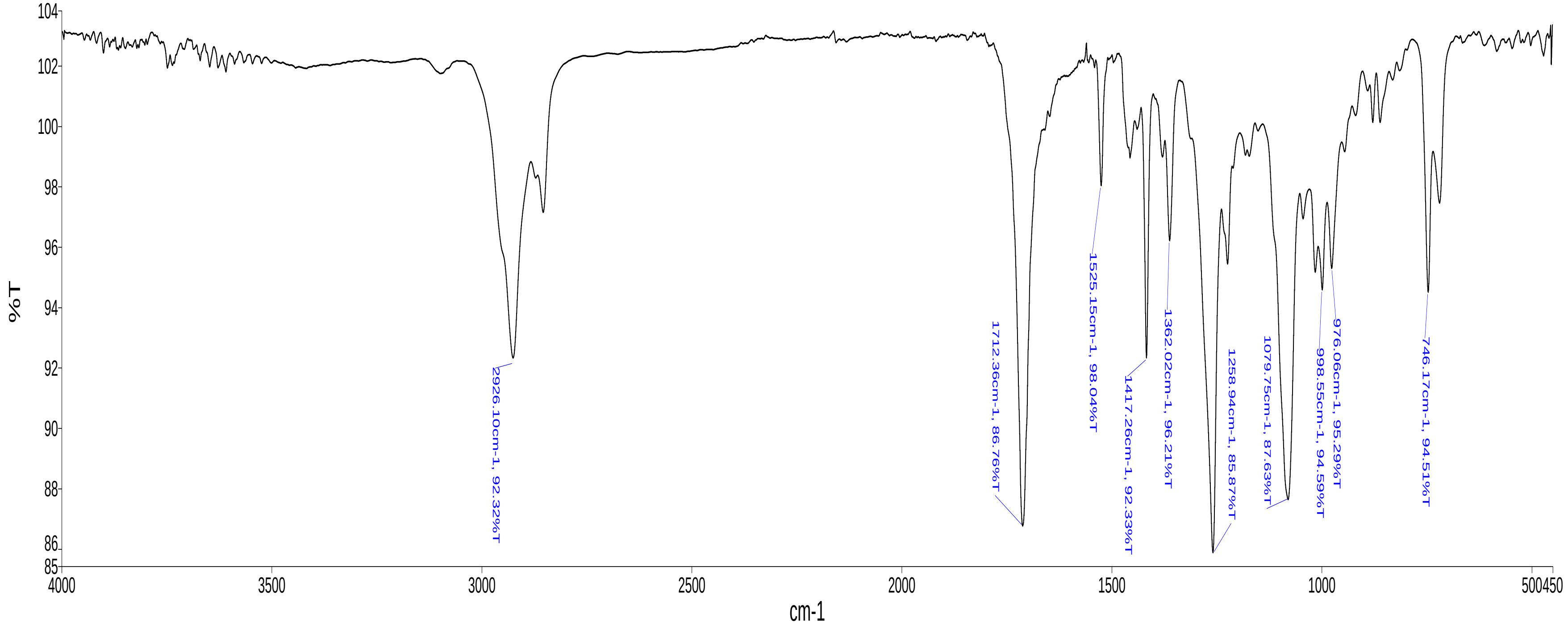

Supplement: Supplementary file 1 [file marinedrugs-21-00158-s001.zip › marinedrugs-2200861-supplementary.pdf]
